# A novel protein cRERE encoded by a circular RNA directly targets ERK signaling to alleviate chemotherapy-induced neuropathic pain

**DOI:** 10.1186/s12964-025-02455-x

**Published:** 2025-10-17

**Authors:** Jian-Bo Zhang, Zhong-Bao Zhao, Jia-Yan Wu, Yu-Juan Duan, Da-Qiang Zhou, Qiong Li, Xu-Han Ren, Xiao-Hua Yang, Yu-Ting Zhao, Shu-Quan Zhao, Mei-Ying Chen, Xiang-Zhong Zhang, Wen-Jun Xin, Guo-Qing Guo, Jing-Dun Xie, Ting Xu

**Affiliations:** 1 Department of Pain Medicine, The Peking University Shenzhen Hospital, Shenzhen, 518036 P. R. China; 2 Guangdong Province Key Laboratory of Brain Function and Disease, Department of Physiology and Pain Research Center, Zhongshan Medical School, Sun Yat-sen University, No. 74, Zhongshan 2nd Road, Guangzhou, 510080 P. R. China; 3 Institute of Neurological Diseases, Shenzhen Bay Laboratory, Gaoke Innovation Center, Shenzhen, 518132 P. R. China; 4 Department of Anesthesiology, Tongji Hospital, Tongji University School of Medicine, Shanghai, 200065 P.R. China; 5 Department of Pain Management, People’s Hospital of Longhua, Shenzhen, 518109 P.R. China; 6 School of Medicine, Xizang Minzu University, Xianyang, 712082 Shaanxi P. R. China; 7 Department of Anesthesiology, State Key Laboratory of Oncology in South China, Guangdong Provincial Clinical Research Center for Cancer, Sun Yat- sen University Cancer Center, No. 651 Dongfeng East Road, Guangzhou, 510060 P. R. China; 8 Faculty of Forensic Medicine, Zhongshan Medical School, Sun Yat-sen University, Guangzhou, 510080 P. R. China; 9 Department of Hematology, The Third Affiliated Hospital of Sun Yat-sen University, Guangzhou, 510000 P. R. China; 10 Neuroscience Laboratory for Cognitive and Developmental Disorders, Department of Anatomy, Medical College of Jinan University, No. 601, Huangpu Avenue West, Guangzhou, 510630 P. R. China

**Keywords:** CircRNA, Coding function, Neuropathic pain, Spinal dorsal horn, Signaling pathway

## Abstract

Circular RNAs (circRNAs), as stable and evolutionarily conserved epigenetic regulators, have attracted growing attention, especially those enriched in the central nervous system (CNS). CNS-specific circRNAs downregulated during disease progression are increasingly recognized as potential therapeutic targets. The clinical translation of circRNAs for stroke treatment further supports the feasibility of circRNA-based therapies, raising the question of whether certain circRNAs may also modulate chemotherapy-induced neuropathic pain (CINP). In this study, we report the identification of a dorsal horn–specific circRere, which is significantly downregulated following vincristine (VCR) administration. Mechanistically, circRere encodes a novel protein, cRERE, in an N6-methyladenosine (m6A)-dependent manner. cRERE alleviates CINP by spatially interfering with the phosphorylation activation site of extracellular signal-regulated kinase 1 (ERK1), thereby preventing downstream activation of the CREB/IL-1β signaling cascade. Taken together, our findings reveal that circRere exerts analgesic effects via an unconventional translation mechanism that generates a functional protein. This study highlights the therapeutic potential of targeting disease-specific downregulated circRNAs and their encoded endogenous proteins for the treatment of CINP.

**Supplementary Information:**

The online version contains supplementary material available at 10.1186/s12964-025-02455-x.

## Introduction

Vincristine (VCR) is a key drug in combination chemotherapy regimens for treating most pediatric cancers, such as acute lymphoblastic leukemia, rhabdomyosarcoma, and neuroblastoma [1]. The main side effect of VCR is peripheral neuropathy, characterized by peripheral and predominantly symmetrical sensory abnormalities, particularly pain hypersensitivity. This significantly limits the drug dosage, delays treatment cycle, and even lead to chemotherapy cessation [2, 3]. However, there is no effective medications for the prevention or treatment of chemotherapy-induced neuropathic pain (CINP).

As a significant epigenetic regulatory mechanism, the characteristic feature of circular RNA (circRNA) is “exon skipping” or “direct back-splicing”, leading to the formation of a covalently closed loop structure without 5’ cap and a 3’ polyA tail, and it is widely expressed throughout the entire eukaryotic transcriptome [4, 5]. Our previous studies, along with those of others, have demonstrated that several circRNAs, including circAnks1a, circFhit, circSCMH1, and circFoxO3, exhibit regionally enriched expression in the central nervous system (CNS) and play important roles in the regulation of CNS functions. Although the relative expression abundance is often lower than that of linear mRNA [6], circRNA is partially resistant to degradation by ribonucleases due to the circular structure, resulting in an average half-life extension of more than 2.5 times compared to linear mRNA transcribed from the same gene [7, 8]. Extensive studies have confirmed that circRNA plays various roles in regulating transcription, translation, and participating in cellular metabolism. Excitingly, researchers have discovered a series of endogenous circRNAs with coding potential in mammals, and proteins translated from these circRNAs can exert diverse biological functions, playing roles in processes such as tumor initiation, proliferation, and metastasis [9]. For instance, circβ-catenin derived from the oncogene β-catenin can translate into the β-catenin-370aa protein, continuously activating the Wnt/β-catenin pathway to promote the growth and migration of liver cancer cells [10]. Additionally, circFBXW7, highly expressed in malignant gliomas, regulates the stability of c-Myc and inhibits the occurrence and metastasis of malignant gliomas by translating the FBXW7-185aa protein [11]. However, the question of the existence of coding circRNAs that are involved in neuropathic pain is a novel topic.

In this study, we identified a circRere, which is significantly downregulated in the spinal dorsal horn following VCR administration in a rat model of CINP. We discovered that circRere encodes a previously uncharacterized protein-cRERE, via an N6-methyladenosine (m6A)-dependent translation mechanism. Functional analyses revealed that cRERE binds to ERK1, inhibits its phosphorylation, and subsequently attenuates activation of the CREB/IL-1β signaling cascade, a key driver of spinal sensitization and neuropathic pain behaviors. Importantly, circRere and cRERE exhibit spinal cord-specific expression and pain-responsive regulation, underscoring their therapeutic specificity and translational potential. Our findings not only highlight a novel coding function for a pain-relevant circRNA, but also point to a broader therapeutic paradigm in which endogenously encoded peptides from ncRNAs may serve as safe, specific, and mechanistically novel agents for treating complex neurological disorders such as CINP.

## Materials and methods

### Animals

Male Sprague-Dawley (SD) rats weighing 200–220 g and aged 6–7 weeks was obtained from the Institute of Experimental Animals of Sun Yat-sen University. All animals were kept at 24 °C and 50–60% humidity under a 12:12-h light/dark cycle and with ad libitum access to food and water. All experimental procedures were approved by the Local Animal Care Committee and were conducted in accordance with the guidelines of the National Institutes of Health (NIH) on animal care and with the ethical guidelines.

### Drug administration

Vincristine (VCR, GC38410, GlpBio Technology Inc., China) dissolved in saline to a concentration of 50 µg/ml was intraperitoneally injected at a dose of 0.5 mg/kg daily for 10 consecutive days. Control animals received an equivalent volume of saline.

### Behavioral tests

Three different forms of stimuli to induce hypersensitivity in rats for behavioral testing paradigms in this study. Prior to the tests, all animals were habituated to the environment by being placed in transparent plastic box for 3 consecutive days (60 min per day) for adaptation.

For von Frey test, von Frey filaments that produce different forces were applied alternately to the plantar surface of the hind paw. In the absence of a paw withdrawal response, a stronger stimulus was presented; when paw withdrawal occurred, the next weaker stimulus was chosen. Optimal threshold calculation by this method required 5 responses in the immediate vicinity of the 50% threshold.

For thermal hyperalgesia, Hargreaves test was conducted using a plantar test apparatus (LifeScience^®^ Plantar Test Apparatus). Briefly, a radiant heat source beneath a glass flour was aimed at the plantar surface of the hind paw. Three measurements of hind paw withdrawal latency were taken for each hind paw and averaged as the result of each test. A 15-s cutoff was set to prevent tissue damage.

For cold allodynia, the acetone evaporation test was performed at the plantar surface of the hind paw. Briefly, 200 µl of acetone is sprayed on the plantar surface of the hind paw, eliciting cooling of the skin to innocuous temperatures of 15–21 ℃. During two minutes after spraying, sensitivity to cold is recorded by quantifying the duration of nocifensive responses. Three measurements of hind paw nocifensive responses were taken for each hind paw and averaged as the result of each test.

All the experiments were performed by investigators who were blinded to the treatments/conditions.

### CircRNA sequencing

The quality and integrity of total RNA samples were rigorously assessed prior to library construction. Agarose gel electrophoresis was performed to evaluate RNA degradation and to detect potential genomic DNA contamination. RNA integrity was determined using the Agilent 2200 Bioanalyzer, which generates electropherogram profiles and calculates the RNA integrity number (RIN). Only samples with a RIN value > 7.0 were considered suitable for downstream applications. Then a total of 2 µg high-quality RNA per sample was subjected to rRNA depletion using the Epicenter Ribo-Zero rRNA Removal Kit (Illumina) and RNase R (Epicenter) to remove linear RNA. Then, RNA-seq libraries were constructed using the NEBNext^®^ UltraTM RNA Library Prep Kit for Illumina^®^ (NEB) according to the manufacturer’s instructions. The libraries were quality controlled with Agilent 2200 TapeStation (Agilent) and sequenced using Illumina HiSeq 3000 by RiboBio Biotechnology Co., Ltd (Guangzhou, China).

### Bioinformatics analysis

Raw sequencing data were first subjected to quality control using standard filtering procedures to remove adapter sequences and low-quality reads, resulting in high-quality clean data. Clean reads were then aligned against a rRNA database (RNAcentral) to eliminate rRNA contaminants, and the remaining reads were defined as effective reads. These effective reads were subsequently mapped to the rat reference genome (Rnor_6.0), and the mapping results were used for circRNA identification. CircRNA prediction was performed by integrating the results from two independent circRNA detection algorithms-CIRI2 and CIRCexplorer2-to identify high-confidence circRNA candidates. A circRNA was considered reliably detected if it was supported by at least one unique back-spliced read in at least one sample. The expression level of each circRNA was calculated using the formula: RPM = (Number of junction reads/Total mapped reads) × 10⁶, followed by annotation and length distribution analysis. Differential expression analysis was conducted using the DESeq package in R, with circRNAs considered significantly differentially expressed if p-value < 0.05 and |log_2_FoldChange| >1.

### RNA Preparation and RT-qPCR

Total RNAs were extracted using TRIzol reagent (15596026, Thermo Fisher Scientific Inc.,USA). For RNase R treatment, 1 µg of RNA was incubated with 1 U of RNase R (Epicenter) for 30 min at 37 °C. Evo M-MLV RT Mix kit (AG11728, Accurate biology Inc., China) and RT Kit with gDNA Clean for Reverse Transcription PCR (AG11705, Accurate biology Inc., China). Quantitative PCR was conducted using SYBR Green Premix Pro Taq HS qPCR Kit (AG11701, Accurate biology Inc., China) on a CFX96 Touch™. The classical ΔΔC_t_ method was used to calculate the relative expression levels of the target genes. Ct values were normalized to the endogenous reference genes (GAPDH for linear RNA and 18 S rRNA for circRNA) as a cDNA loading control, and changes were calculated relative to controls. To specifically detect circRNAs, divergent primers spanning the back-spliced junction were designed and validated. Primer sequences are listed in Supplementary Table 1.

### RNA stability assay

Briefly, 20 µl 2.5 µg/ml ActD was intrathecally injected to inhibit transcription, and the spinal dorsal horn were collected 24 h after ActD treatment for quantification.

### Methylated RNA Immunoprecipitation (meRIP)

MeRIP assay was conducted based on the instruction manual of RIP kit (17–700, Merck, Germany). Briefly, the antibody target to methylated RNA or negative IgG was combined with A/G beads at room temperature for one hour. Afterwards, RNA in equal amount was added into each tube with antibody combined A/G beads. The mixture was incubated at 4 ℃ overnight. The next day, the mixture was washed three times. At last, RNA was extracted from washed beads and analyzed with qRT-PCR.

### Intrathecal or intraspinal injection and AAV construction

A polyethylene-10 catheter was implanted into the L5/L6 intervertebral subarachnoid space after the intraperitoneal injection of sodium pentobarbital (50 mg/kg); the localization of the tip of the catheter was between the levels of the L4-L6 spinal segments. The rats were allowed to recover for 5 days. Animals that exhibited hind limb paresis or paralysis were excluded from the study.

For intraspinal injection of the AAV virus, the L4-L5 vertebrae were exposed, and the vertebral column was mounted in a stereotaxic frame. A slight laminotomy was performed, and the dura was incised to expose the spinal cord. AAV virus was injected into both sides of the spinal dorsal horn at 4 injection sites (200 nl of AAV was injected at each site at a slow rate of 30 nl/min). The micropipette was withdrawn 10 min after viral injection, and the incision was closed with stitches.

The artificail circRere used for intrathecal injection were otained from GENESEED Co.Ltd (Shenzhen, China). 5 µg circRere mixed with 10% glucose solution, Entranster^TM^-in vivo regent (Engreen Biosystem Co, Ltd., Beijing, China) and RNaseR-free sterile water to a total volume of 10 µl, and then intrathecally injected into one rat. The cRERE protein used for intrathecal injection were obtained from Sangon Biotech Co., Ltd. (Shanghai, China) and HUABIO Co. Ltd (Hangzhou, China). 5 µg cRERE was dissolved in normal saline a total volume of 10 µl, and then intrathecally injected into one rat. circRere and cRERE were intrathecally injected once, ten days after the first dosage of VCR, and behavioral test was conducted accordingly. The dosage of circRere and cRERE was determined based on our previously behavioral data (data not shown).

Adeno-associated virus 2/9 (AAV2/9) were constructed using standard molecular cloning techniques with assistance from Obio Technology (Shanghai, China), Sunbio (Shanghai, China), or BrainVTA (Wuhan, China). The AAV constructs carried circRere, circRere or shRNA under the control of the hSyn promoter. Control vectors expressing GFP only or scrambled sequence were constructed in parallel. The final AAV vectors were packaged into serotype 2/9 with titers ranging from 1 × 10¹³ vg/ml and used for in vivo injection. Lentivirus (LV) were designed and constructed by standard methods with assistance from BrainVTA (Wuhan, China).

### Spinal cord slice preparation

The L4-L6 spinal cord was quickly removed from the lumbar vertebrae and transferred to oxygenated (95% O_2_ and 5% CO_2_) ice-cold slice solution containing (in mM): 126 NaCl, 3 KCl, 10 D-glucose, 26 NaHCO_3_, 1.2 NaH_2_PO4, 0.5 CaCl_2_, and 5 MgCl_2_. The dorsal and ventral roots were carefully removed except that in some slices the associated dorsal roots were kept for reception of stimulation from the stimulus isolator. The spinal cord was coated with agarose (Sigma, USA), and 400-µm thick acute spinal L4-L6 cord slices were cut on a vibratome (Leica VT-1000 S). The slices were incubated in continuously oxygenated standard artificial cerebrospinal fluid (ACSF: 125mM NaCl, 3mM KCl, 26mM NaHCO_3_, 1.25mM NaH_2_PO4, 2mM CaCl_2_, 1mM MgCl_2_ and 10mM D-glucose, pH 7.3) for at least 1 h at 33 °C and then transferred to the recording chamber. Lamina I to II neurons were visualized using a 40× water-immersion objective on an upright infrared Nikon microscope (Nikon, Japan).

### Whole-cell recordings

The recording chamber was continuously perfused with pre-heated 33 °C ACSF at a rate of 2 ml/min. The pipettes (3-6MΩ, ~ 2 μm tip diameter) were pulled from borosilicate glass with filament (OD: 1.2 mm, ID: 0.69 mm) on a P-2000G micropipette puller (Sutter Instruments, USA). In the experiments involving recording of evoked responses, a concentric bipolar electrode (FHC, Bowdoin, ME USA) connected to a constant-current stimulus isolator (DS3; Digitimer Ltd., UK) was used to stimulate the spinal cord dorsal root. Data were recorded using an EPC 10 amplifier (HEKA Elektronik, Germany). Stimulus delivery and data acquisition were performed using Patchmaster software (HEKA Elektronik). A seal resistance of ≥ 2GΩ and an access resistance of ≤ 20MΩ were considered acceptable. The electrophysiological data were replaced and analyzed by Clampfit10.4 (Axon Instruments Inc., USA) and miniAnalysis program6.0.7 (Synaptosoft Inc., Decatur, GA, USA).

### C-fiber-evoked action potentials in vivo

C-fiber–evoked field potentials in the spinal dorsal horn were recorded. The left sciatic nerve of rats was dissected free for bipolar electrical stimulation. C-fiber-evoked responses in the spinal dorsal horn (L4-L6 segments) were recorded at a depth of 50–500 μm from the dorsal surface with a microelectrode (impedance, 1–2 MΩ; exposed tip diameter, 1–2 μm). The distance from the stimulation site at the sciatic nerve to the recording site in the lumbar spinal dorsal horn was approximately 11 cm. A bandwidth of 0.1–500 Hz was used to record field potentials. An A/D converter card (DT2821-F-16SE, Data Translation) was used to digitize and store data in a Pentium computer at a sampling rate of 10 kHz. Single square pulses (0.5 ms duration at 1 min intervals) delivered to the sciatic nerve were used as test stimuli. The amplitude of action potentials was counted at different simulation intensities.

### Co-immunoprecipitation (Co-IP)

Co-IP was conducted using a Co-Immunoprecipitation Kit (88804, Pierce™, USA). Spinal dorsal horn tissues were excised quickly and placed in lysis buffer. Total protein was extracted and determined the concentration with BCA assay. Cell lysate, containing 1000 µg of total protein, was combined with 10 µg of IP antibody per sample in a microcentrifuge tube. This antigen sample/antibody mixture was incubated overnight at 4 °C to form the immune complex. The next day, 0.25 mg of Pierce Protein A/G Magnetic Beads was washed twice with IP Lysis/Wash Buffer. The previous antigen sample/antibody mixture was mixed with pre-washed magnetic beads and incubated at room temperature for 1 h with mixing. The beads with a magnetic stand were collected and wished twice with IP Lysis/Wash Buffer and ultra-pure water. The unbound sample was removed and save for analysis. The washed beads were eluted with Elution Buffer at RT with mixing for 10 min. The beads were magnetically separated and saved the supernatant containing the target antigen. To neutralize the low pH of the immune complexes, Neutralization Buffer was added. The immune complexes were analyzed by western blotting.

### Liquid chromatography-mass spectrometry (LC-MS) analysis

The Co-IP immune complexes were digested with trypsin (Promega). The digested peptides fragments were analyzed on an EASY-nLC1200 couple to Q Exactive (Thermo Scientific). The mass spectrometry data were analyzed and identified using MaxQuant software(2.0.1.0)and the UniProt Rattus database.

### RNA fluorescence in situ hybridization (FISH) and Immunofluorescence

Animals were perfused through the ascending aorta with 4% paraformaldehyde under anesthesia.

For FISH, the spinal cord tissues were cut into 25 µm-thick transverse sections after 30% DEPC-sucrose dehydration at 4 °C and hybridized at 42 °C for 18 h with the 3’ and 5’-FAM-labeled circ-Rere probe 5’-ATTCAGCCTCCACTTCATCCTC-3’ (1:100 dilution, EXQON). Subsequent to FISH, immunofluorescence staining was performed to assess the co-localization of circRere with cellular markers. The combined procedure enables visualization of circRere subcellular distribution in relation to specific cell types.

For Immunofluorescence, spinal cryostat Sect. (25 μm thick) were cut and blocked with 1% BSA for 1 h at room temperature. Sections were then incubated overnight at 4 °C with primary antibodies against cRERE (Customized, HUABIO, China), NeuN (MAB377, Millipore, USA), GFAP (3670, CST, USA), Iba1 (ab5076, Abcam, UK), CaMKII (ab52476, Abcam, UK), PAX2 (AF3364, R&D Systems, USA). On the following day, sections were incubated with Cy3, Alexa 488-conjugated secondary antibody at room temperature for 1 h. The stained sections were imaged using a Nikon (ECLIPSE Ni-E) florescence microscope equipped with 20X/0.75NA and 40X/0.85NA objective lenses. Z-stack imaging (10 μm total thickness) was performed to ensure accurate assessment of subcellular co-localization and maximum intensity projections were used for image display.

### Dual-luciferase reporter assay

To test the biological activity of the predicted internal ribosome entry site (IRES) in circRere, we constructed a bicistronic reporter plasmid by inserting the candidate IRES sequences into the intercistronic region between the Renilla luciferase (Rluc) and firefly luciferase (Luc) coding sequences. The insertion was carried out using KpnI and EcoRI restriction enzyme sites in a modified dual-luciferase reporter backbone (based on the pRF vector). The IRES of hepatitis C virus (HCV) was used as a positive control, while an empty vector without an inserted IRES element served as a negative control. HEK293T cells were seeded in 24-well plates and transfected with 1 µg of the reporter plasmid using Lipofectamine 2000 (Invitrogen) according to the manufacturer’s protocol. After 48 h, cells were harvested and luciferase activities were measured using a Dual-Luciferase^®^ Reporter Assay System (TransGen™). The results were normalized as the ratio of firefly to Renilla luciferase activity (Luc/Rluc) to reflect IRES-mediated translation efficiency.

### CRISPR vector of specifically targeting m6A site in circrere

The dCasRx and fat mass and obesity-associated protein (FTO) fusion vector construction in Lenti-CRISPR was conducted as the previously described with few modifications [12]. Briefly, full-length of Fto/Alkbh5 was amplified from rat cDNA and then ligated into the BamHI treated EF1a-dCasRx-2 A-EGFP vector with ClonExpress II One Step Cloning kit (C112, Vazyme). Lentivirus guide RNA (LV-gRNA, sequence of gRNA-GCAAATCTGGAAGTTTGGCCTGA) and negative control gRNA were constructed by BrainVTA (Wuhan, China). Lentivirus-dCasRx-FTO was also constructed by BrainVTA (Wuhan, China). LV-gRNA and LV-dCasRx-FTO were co-intraspinal injection into L4-L6 spnial cord and then used in the experiment after the identification of their effect.

### Western blotting

Proteins obtained from spinal dorsal horn tissues were separated by gel electrophoresis SDS-PAGE and transferred to a PVDF membrane. The PVDF membrane was incubated first with Protein Free Rapid Blocking Buffer (PS108P, epizymeTM, China) for one hour. Then the PVDF membrane was incubated with primary antibodies against cRERE (Customized, HUABIO, China), phospho-ERK1/2 (ET1610-13, HUABIO, China), ERK1/2 (ET1601-29, HUABIO, China), phospho-CREB (R23982, Zenbio, China), CREB (ET1601-15, HUABIO, China), phospho-STAT3 (sc-8059, Santa Cruz Biotechnology, USA), STAT3 (#12640, CST, USA), c-fos (EM1710-29, HUABIO, China), IL-1β (ab283818, abcam, UK) or GAPDH (EM1101, HUABIO, China) overnight at 4 °C. The blots were then incubated with secondary antibodies conjugated to horseradish peroxidase. The immuno-stained bands were quantified using a computer-assisted imaging analysis system (ImageJ). All uncropped images for full-length blots and gels were presented in the Source Data file.

### Molecular docking

The 3D structure of ERK1 was downloaded from RCSB Protein Data Bank (PDB ID: P21708). Protein–protein docking in ClusPro server 2.0 (https://cluspro.org) [13] was used for molecular-docking simulations of proteins. Molecular graphics were generated using PyMOL.

### Polysome isolation and analysis by RT-qPCR

PC-12 cells were grown in complete REMI 1640 medium to 80% confluency. Following quick aspiration of media, plates were placed on top of liquid nitrogen and immediately transferred to ice. Three milliliters nuclease-free PBS with 100 mg/mL cycloheximide was added and cells were scraped into PBS. The mixture was centrifuged at the speed of 2000 rpm under 4 ℃ for 5 min. The supernatant was removed and lysis buffer (3 mL, 10 mM Tris-HCl [pH 7.4], 5 mM MgCl_2_, 100 mM KCl, 1% Triton X-100, 2 mM DTT, 100 mg/mL cycloheximide) was added. Cell lysates were passed through a 26-G needle 10 times and clarified by spinning at 20,000 x g for 10 min at 4℃. The supernatant was moved into a new, nuclease-free tubes and stored at −80℃ until the next step. 10%−50% sucrose gradient was prepared in gradient buffer (0.5 M Tris-acetate [pH 7.0], 0.5 M NH4Cl, 0.12 M MgCl2) using a BioComp Gradient Master and spun at 38,000 rpm in a SW 41 Ti rotor for 3 h at 4℃. Fractionation, recording of 260 nm absorbance and fractions collection were performed using a Biocomp Density Gradient Fractionation system. RNA from each of 12 collected fractions was ethanol precipitated overnight at −80℃. Pelleted RNA was resuspended in LET (25 mM Tris-HCl, pH 8.0, 100 mM LiCl, 20 mM EDTA, pH 8.0) and SDS to 1%, extracted twice with phenol/chloroform/LET, and then ethanol precipitated using NH4OAc and 1 mL Glycoblue (ThermoFisher Scientific AM9515). The precipitated RNA was analysis with RT-qPCR as previous described in the RNA preparation and RT-qPCR section.

### Statistics

Statistical analysis was carried out using Microsoft Excel 2016, GraphPad Prism v.9.00 for Windows and SPSS v.25.0. Experimental data are represented as the average ± SEM of a minimum of three biological replicates. The normally distributed data were analyzed using the two independent samples t-test or one-way ANOVA followed by Dunnett’s T3 post hoc test. When tests of normality were not satisfied, the non-parametric test (Mann-Whitney test) was substituted. The data on pain behavior were analyzed using one-way repeated-measures ANOVA. The criterion of statistical significance was 0.05. To minimize the number and suffering of animals as much as possible, the sample size was determined according to previous publications in behavioral and pertinent molecular studies. All measurements were taken from distinct samples.

## Results

### Downregulation of coding-poteintial circrere involved in vincristine-induced neuropathic pain

Based on our finding and those of peers, VCR-induced neuropathic pain is a well-established model of CINP [14, 15]. In this study, VCR-treated rats exhibited significant mechanical allodynia, heat hyperalgesia, and cold allodynia compared to vehicle controls (Fig. S1a-c). Since nociceptive C-fiber from the first synapses with dorsal horn neurons, we assessed C-fiber-mediated synaptic transmission by recording spinal C-fiber-evoked field potentials in vivo. As shown in Figure S1d, both groups showed increased field potential amplitudes with rising stimulation intensity (4 V and 14 V), and the VCR group displayed an up-left shift in the stimulus-response curve, indicating enhanced C-fiber synaptic transmission. Patch-clamp recordings further revealed significantly increased amplitude and frequency of miniature excitatory postsynaptic currents (mEPSCs) in the spinal dorsal horn neurons on day 20 post-VCR treatment (Fig. S1e). Additionally, depolarization-induced neuronal firing also was markedly elevated in neurons (Fig. S1f). In summary, these findings indicated that VCR enhances spinal dorsal horn central sensitization and promotes neuropathic pain, highlighting the importance of developing effective preventive and therapeutic strategies targeting this mechanism.

**Fig. 1:**
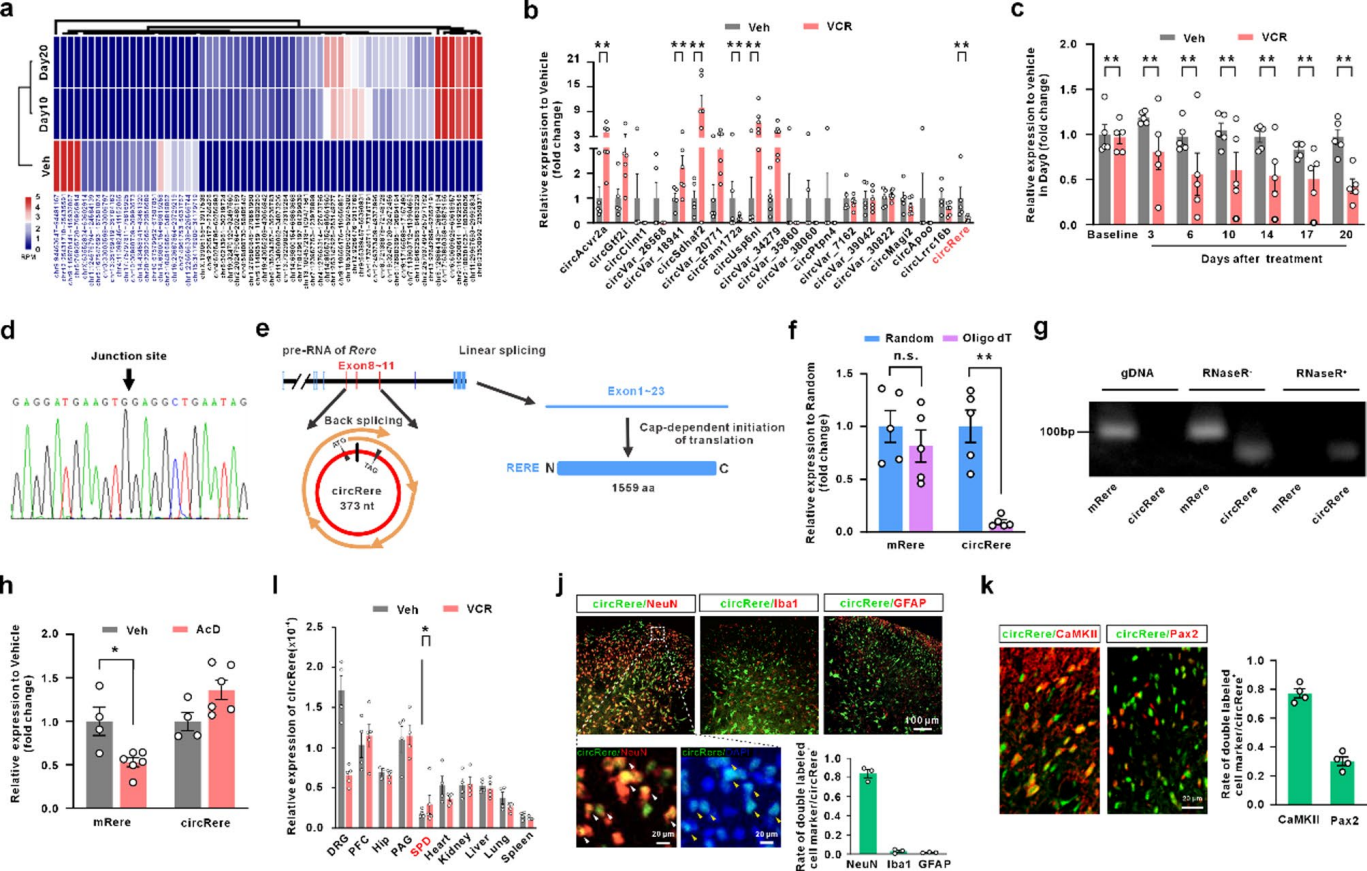
CircRere downregulated in the spinal dorsal horn nurons following Vincristine treatment. **a** The heatmap showed the DEcRs in the dorsal horn after 10 and 20 days of VCR treatment compared to the vehicle group. **b** The expression level of 20 DEcRs, with an ORF spanning the circular junction at least once, was confirmed by qPCR validation (** *P* < 0.01 vs. the vehicle group, Mann-Whitney test, *n* = 5 for vehicle group and *n* = 6 for VCR group). **c** The expression level of circRere in the time course of the VCR rat model was detected. (** *P* < 0.01 vs. the vehicle group, Student’s t-test, *n* = 5 in each group,). **d** The sequence containing junction site detected in Sanger sequencing. The arrow pointed out the junction site. **e** The splice and predicted translation mode was drawn based on the Sanger sequencing and silico analysis. **f** The circular characteristics of circRere was validated via random hexamer or oligo(dT)_18_ primers used in reverse transcription experiments. (** *P* < 0.01 vs. the random hexamer primer group, Student’s t-test, *n* = 5 in each group,). **g** PCR showed that dorsal horn circRere, but not mRere, was resistant to digestion by RNaseR. **h** The half-life test of circRere and mRere via treatment with actinomycin D, a chemical inhibiting transcription (vs. the vehicle group, Student’s t-test, *n* = 5 for vehicle group and *n* = 7 for AcD group). **i** The expression of circRere in different tissues was examined of vehicle and VCR treatment rats (* *P* < 0.05 vs. the vehicle group, Mann-Whitney test, *n* = 4 for vehicle group and *n* = 5 for VCR group). **j** CircRere mostly expressed in neurons (NeuN-positive cells), but not microglia (Iba1-positive cells) or astrocyte (GFAP-positive cells), in spinal dorsal horn (*n* = 3 in each group). **k** CircRere primaly co-localized with CaMKII-positive neurons in dorsal horn, while a small mount co-localized with Pax2-positive neurons (*n* = 4 in each group)

With the expanding frontiers of biomedical research, hundreds of functional peptides or proteins have been identified as being translated from non-coding regions of the genome, including long intergenic non-coding RNAs (lincRNAs), circRNA, 5′ untranslated regions (5′-UTRs), and pri-microRNAs [16, 17]. However, the coding mechanisms of circRNAs and the potential therapeutic roles of their encoded products in various diseases remain to be elucidated. Building upon previous methodologies [18, 19], we generated a circRNA expression profile from a rat model of VCR-induced neuropathic pain using RNA sequencing of ribosomal RNA-depleted and RNase R-treated samples. Differential expression analysis of spinal dorsal horn tissue on days 10 and 20 post-VCR administration identified 62 differentially expressed circRNAs (DEcRs), including 41 upregulated and 21 downregulated circRNAs (Fig. 1a, Fig. S2a). From the downregulated circRNAs, we screened for candidates containing an open reading frame (ORF) spanning the back-splice junction and exhibiting sustained suppression after VCR treatment (Tables S2 and S3). This led to the identification of circRere (circ: chr5:167547693–167581074) (Fig. 1b, c). In silico analysis indicated that circRere is derived from exons 8–11 of the *Rere* gene (373 bp), which was validated by Sanger sequencing (Fig. 1d, e; Fig S2b). To confirm its circular nature, reverse transcription with random hexamer or oligo (dT)_18_ primers revealed that oligo (dT)_18_ failed to amplify circRere, but not linear Rere mRNA (mRere) (Fig. 1f). CircRere also showed RNase R resistance (Fig. 1g), and a longer half-life (>24 h) than mRere following actinomycin D treatment in vivo (Fig. 1h), confirming its stability as a circular RNA. Expression analysis showed that circRere is predominantly enriched in the spinal dorsal horn under physiological conditions, but is significantly reduced after VCR treatment, implicating its involvement in VCR-induced neuropathic pain (Fig. 1i). FISH using the validated circRere-specific probe revealed that circRere localized to both the nucleus and cytoplasm of dorsal horn neurons, but not in astrocytes or microglia. Notably, over 80% of circRere signal co-localized with CaMKIIα-positive excitatory neurons (and Fig. 1l, k). Specificity validation of the circRere probe is presented in Figure S3a. Sequence alignment with circBase identified five homologous circRNAs derived from the human *RERE* gene (Table S4), suggesting that circRere and its encoded product may be evolutionary conserved and represent a promising target with translational potential.

### CircRere encodes a novel protein cRERE in a m6A-dependent manner

**Fig. 2:**
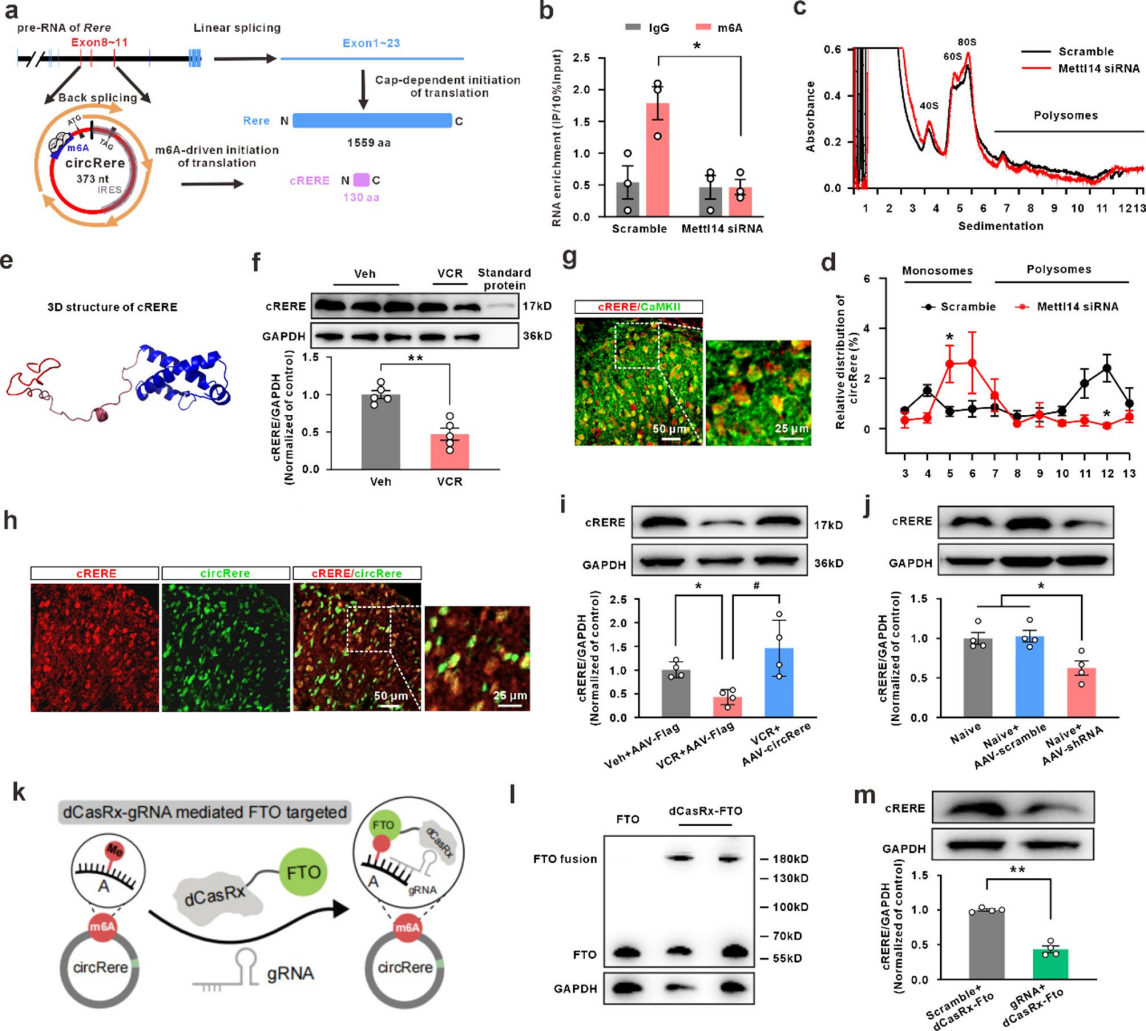
CicRere translates the protein cRERE through m6A-mediated mechanisms. **a** The translation model was drawn based on the Sanger sequencing and silico analysis. The red circle was represented as circRere. The gray line on the circRere was the predicted IRES sequence and the blue line under the ribosome was the predicted high confidential m6A site. **b** MeRIP was conducted and circRere was detected subsequently (* *P* < 0.05 vs. the scramble group, Student’s t-test, *n* = 3 in each group). **c** The representation of polysome profiling of PC-12 cells with siRNA or scramble treatment. **d** Relative levels of circRere in each ribosome fraction were quantified by qPCR (* *P* < 0.05 vs. the scramble group, Student’s t-test, *n* = 3 for scramble group and *n* = 5 for Mettl14 siRNA group). **e** Three-dimensional structural model of cRERE predicted by AlphaFold2. **f** The western-blotting detection of the specific rabbit polyclonal antibody with the synthetic protein (** *P* < 0.01 vs. the vehicle group, Student’s t-test, *n* = 5 in each group). **g** cRERE primaly co-localized with CaMKII-positive neurons in dorsal horn (*n* = 3). **h** In situ hybridization histochemistry combined with immunofluorescence technique showed the co-localization of cRERE and circRere-positive cells in naïve rats (*n* = 3). **i** The western-blotting detection of spinal dorsal horn from rats with intraspinal injection of AAV-FLAG and AAV-hSyn-circRere (* *P* < 0.05 vs. the veh + AAV-FLAG group, # *P* < 0.05 vs. the VCR + AAV-FLAG group, one-way ANOVA, *n* = 4 in each group). **j** The western blotting detection of spinal dorsal horn from rats with intraspinal injection of AAV-scramble and AAV-circRere shRNA-EGFP (* *P* < 0.05 vs. the Naïve or Naive + AAV-scramble group, one-way ANOVA, *n* = 4 in each group). **k** Schematic diagram of dCasRx-gRNA mediated FTO “erasure” m6A to the modification regione in circRere. **l** Identification of dCasRx-FTO fusion protein expression on day 5 after intraspinal injection of CRISPR-dCasRx-Fto. **m** The expression of cRERE decreased significantly after targeting the erasure of m6A modification on circRere using the CRISPR-dCasRx-gRNA system (* *P* < 0.05 vs. the scramble + dCasRx-Fto group, Student’s t-test, *n* = 4 in each group)

Based on the presence of a cross-junction and multiple-round ORF sequence spanning the translation start codon and the stop codon, circRere may encode a 130-amino acid protein. We named this product as a circRNA-encoded RERE (cRERE). To investigate the mechanism by which circRere initiates translation. Our in-silico analysis.

revealed the presence of a potential internal ribosome entry site (IRES) and a potential N6-methyladenosine (m6A) modification site within the circRere sequence (Fig. 2a), indicating that both above classical translation initiation mechanisms could potentially initiate its translation. We first reconstructed the target IRES sequence into a plasmid vector, by using a dual-luciferase reporter gene assay, observed in PC-12 cell line that there was no significant enhanced fluorescence signal compared to the control vector after transfection (Fig. S3b). This indicates that the IRES sequence in circRere lacks translation activity. Then we performed MeRIP on both PC-12 cell line and spinal dorsal horn tissue, the result showed that the predicted sequence of m6A modification in circRere was enriched by m6A-specific antibody. The level of m6A modification was significantly disrupted after m6A methyltransferase-methyltransferase-like protein 14 (Mettl14) siRNA treatment (Fig. S3c and Fig. 2b), suggesting that the predicted circRere translation start site possessed an m6A modification. Subsequently, polysome profiling assay was performed to verify whether the translational initiation of circRere is started by m6A modification. The results showed that the use of Mettle14 siRNA led to changes in the ribosome binding distribution on circRere, shifting from binding polysomes to polysome subunits (Fig. S3d, Fig. 2c, d). Validation of Mettl14 siRNA efficiency by qPCR and western blotting is shown in Figure S4a and S4b.

To further verify that circRere encodes a novel protein, cRERE, we employed AlphaFold2 to predict its three-dimensional structure (Fig. 2e). A polyclonal antibody targeting the cRERE sequence was subsequently generated (Table S5). Using this antibody, western blotting analysis detected a specific protein band at approximately 17 kDa, consistent with the predicted molecular weight (Fig. S3e and Fig. 2f). The immunofluorescence staining results showed that cRERE was in the dorsal horn CaMKIIα-positive neurons and colocalized with circRere (Fig. 2g, h). Furthermore, application of AAV-hSyn-circRere-Flag significantly prevented the decrease of cRERE in the spinal dorsal horn induced by VCR (Fig. 2i). Conversely, intraspinal injection of AAV-hSyn-circRere shRNA-EGFP decreased the expression of cRERE (Fig. 2j). The qPCR validation of AAV-hSyn-circRere overexpression and circRere knockdown via AAV-hSyn-circRere-shRNA-EGFP is presented in Figure S4c and S4d. We used the CRISPR gene-editing system [12, 20] to further confirm the specific regulatory role of m6A in circRere translational initiation. A m6A demethyltransferase-fat mass and obesity-associated protein (FTO) was fused with inactivated CasRx protein (dCasRx/FTO fusion protein) to specifically “erasure” m6A on the motif (+ 357 to + 361) via guide RNA (gRNA)(Fig. 2k). The dCasRx/FTO fusion protein was detectable on day 5 after intraspinal of lentivirus CRISPR-dCasRx-Fto in naïve rats (Fig. 2l), and the m6A level of circRere was decreased by 86.2% compared with the sramble group (Fig. S4e). Importantly, this led to a significant reduction in the expression of cRERE translated from circRere (Fig. 2m).

### cRERE translated from circrere attenuates spinal central sensitization and pain behaviors induced by vincristine

**Fig. 3:**
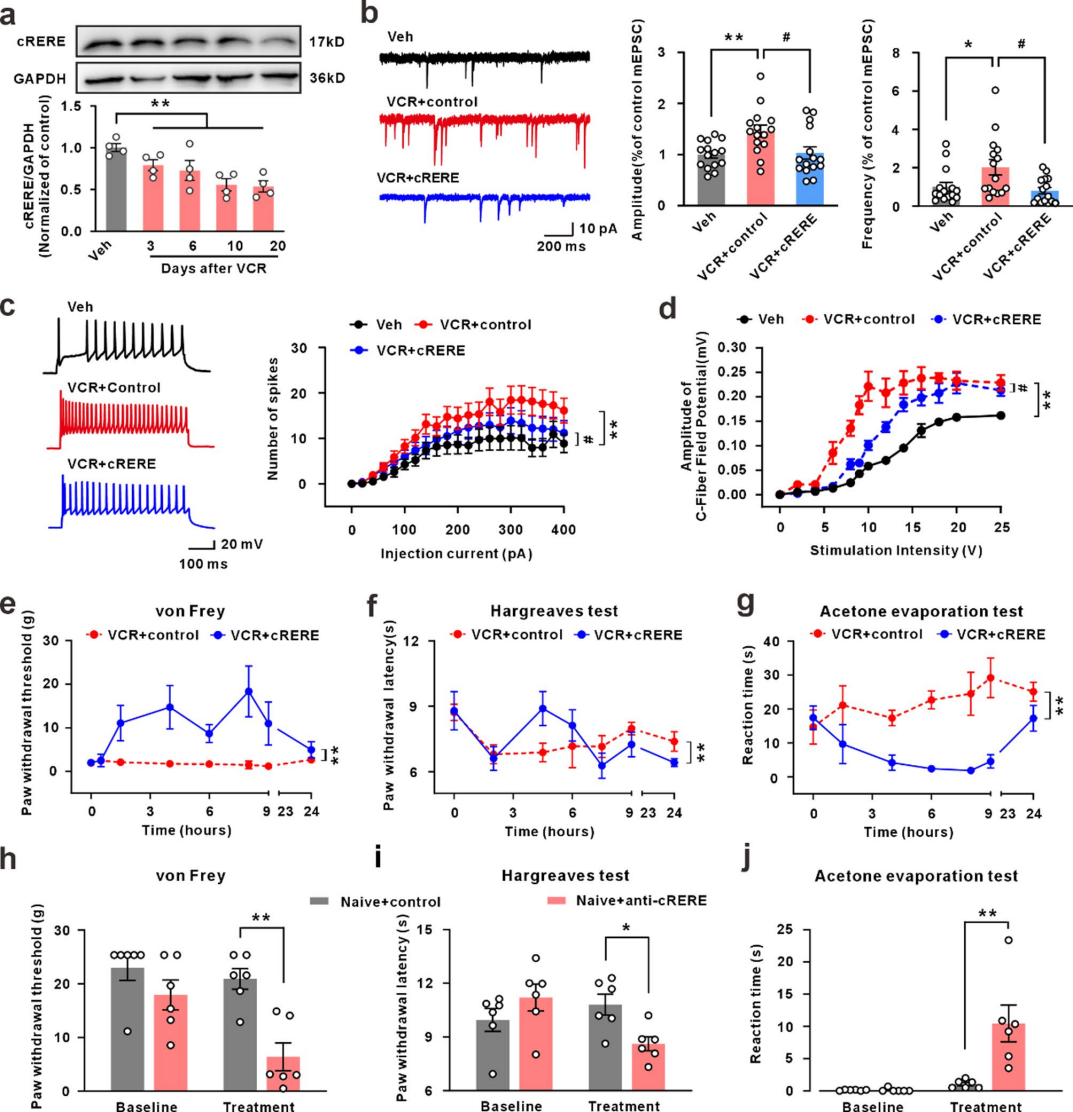
The decreased cRERE in the dorsal horn contributed to the central sensitization and CINP. **a** The expression of cRERE protein on day 3, 6, 10 and 20 flowing VCR-treatment. (** *P* < 0.01 vs. the vehicle group, one-way ANOVA, *n* = 4 in each group). **b** and **c** The ex vivo electrophysiology recording of spinal dorsal horn slice from rats treated by cRERE, but not boiled cRERE, ameliorated the increased amplitude and frequency of neuronal mEPSCs and the number of neuronal firing induced by VCR (* *P* < 0.05 and ** *P* < 0.01 vs. the vehicle group, # *P* < 0.05 vs. the VCR + control group, one-way ANOVA, *n* = 15 cells from 3rats in each group). **d** Stimulus-response curve from in vivo C-fiber evoked potential recording (** *P* < 0.01 vs. the vehicle group, # *P* < 0.05 vs. the VCR + control group, one-way repeated-measures ANOVA, *n* = 5 in each group). **e**-**g** The three behavioral test of rats (von Frey, Hargreaves test, and Acetone evaporation test) with VCR-treatment and intrathecal injection of cRERE (** *P* < 0.01 vs. the VCR + control group, ne-way repeated-measures ANOVA, von Fray: *n* = 8 in each group, Hargreaves test: *n* = 9 in each group, Acetone evaporation test: *n* = 6 for VCR + control group and *n* = 8 for VCR + cRERE group). **h**-**j** The behavioral test of rats with VCR-treatment and intrathecal injection of control or cRERE (*P* < 0.05 and ** *P* < 0.01 vs. the Naïve + control group, Student’s t-test, von Fray: *n* = 6 in each group, Hargreaves test: *n* = 6 in each group, Acetone evaporation test: *n* = 6 in each group)

Next, we examined the role of cRERE in the pain pathological process involved in VCR treatment. Western blotting results showed that the expression of cRERE in the spinal dorsal horn continued to decrease after VCR administration (Fig. 3a). The electrophysiological results from spinal cord slices revealed that incubation with chemically synthesized cRERE significantly improved the increased frequency and amplitude of VCR-induced excitatory neuronal mEPSCs and the increasing of neuronal firing rate (Fig. 3b, c). In vivo electrophysiological results also showed that a down-right shift of the stimulus-response curve was evident in the VCR + cRERE group, compared with the control group (Fig. 3d). Importantly, intrathecal injection of cRERE significantly attenuated the pain behavior of rats induced by VCR (Fig. 3e-g). On the other hand, intrathecal injection of anti-cRERE antibody (0.2 mg/kg) induced pain hypersensitivity in normal rats (Fig. 3h-j), but had no effect on the expression of circRere (Fig. S4f). These results suggested that cRERE encoded by circRere, plays a crucial role in CINP.

**Fig. 4:**
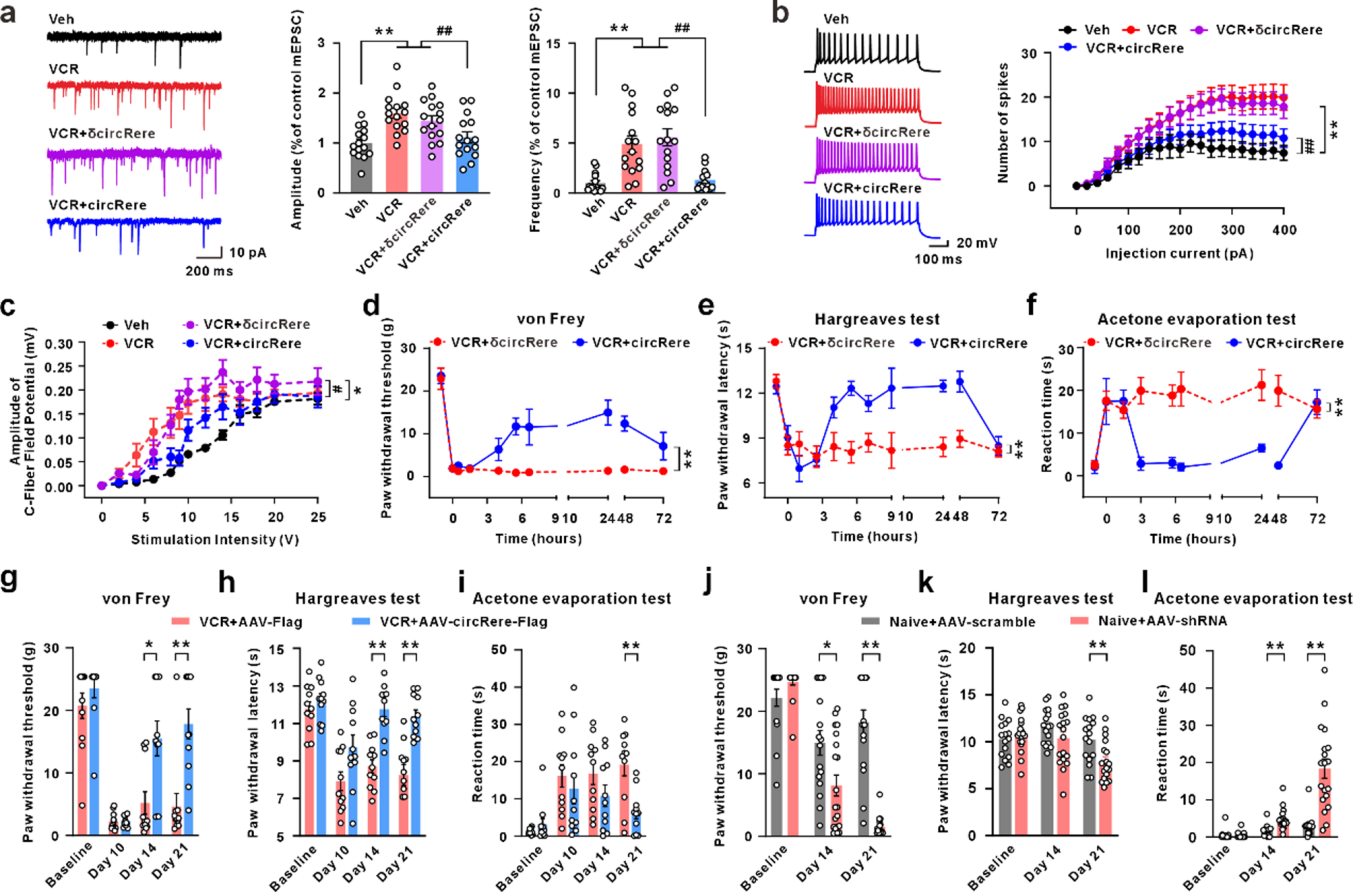
Down-regulation of circRere-mediated central sensitization and pain hypersensitivity following VCR treatment. **a** The frequency and amplitude of mEPSCs recording of spinal dorsal horn from Veh, VCR, VCR + δcircRere and VCR + circRere treated rats (** *P* < 0.01 vs. the vehicle group, ## *P* < 0.01 vs. the VCR/VCR + δcircRere group, one-way ANOVA, *n* = 15 cells from 3 rats). **b** The neuronal firing recording of spinal dorsal horn from Veh, VCR, VCR + δcircRere and VCR + circRere treated rats (** *P* < 0.01 vs. the vehicle group, ## *P* < 0.01 vs. the VCR/VCR + δcircRere group, one-way ANOVA, *n* = 16 cells from 3 rats in vehicle group, *n* = 15 cells from 3 rats in VCR group, *n* = 17 cells from 3 rats in VCR + δcircRere group, *n* = 18 cells from 3 rats in VCR + cRERE group). **c** The stimulation-respond curve of C-fiber-evoked field potential in the spinal dorsal horn (* *P* < 0.05 vs. the vehicle group, # *P* < 0.01 vs. the VCR/VCR + δcircRere group, one-way repeated-measures ANOVA, *n* = 5 for Veh and VCR + circRere group, *n* = 6 for VCR and VCR + δcircRere group). **d**-**f** Behavioral testing results for mechanical allodynia, thermal hyperalgesia and cold allodynia, after intrathecal injection of δcircRere or circRere in VCR rats (** *P* < 0.01 vs. the VCR + δcircRere group, one-way repeated-measures ANOVA, *n* = 6 in each group). **g**-**i**. Behavioral testing results for mechanical allodynia, thermal hyperalgesia and cold allodynia, after intraspinal injection of recombinant AAV- Flag or AAV-circRere-Flag in VCR rats (** *P* < 0.01 vs. the VCR + AAV-Flag group, one-way repeated-measures ANOVA, *n* = 11 in each group). **j**-**l**. Behavioral testing results for mechanical allodynia, thermal hyperalgesia and cold allodynia, after intraspinal injection of recombinant AAV-scramble or AAV-shRNA in naïve rats (* *P* < 0.05 and ** *P* < 0.01 vs. the Naive + AAV-scramble group, one-way repeated-measures ANOVA, *n* = 16 for AAV-scramble, *n* = 20 for AAV-shRNA in von Frey test, *n* = 16 for AAV-scramble, *n* = 18 for AAV-shRNA in Acetone evaporation test, *n* = 18 for AAV-scramble, *n* = 19 for AAV-shRNA in Hargreaves test). AAV-Flag represents AAV-hSyn-Flag, AAV-circRere-Flag represents AAV-hSyn-circRere-Flag, AAV-scramble represents AAV-hSyn-scramble-EGFP, AAV-shRNA represents AAV-hSyn-circRere shRNA-EGFP

To further verify that circRere alleviates VCR-induced spinal central sensitization and pain behaviors through encoding cRERE, we chemically synthesized two constructs based on the previously identified nucleotide sequence: one containing a normal start codon (ATG, + 366 to + 368, first nucleotide on the right side of the junction site as shown in Fig. S1b was designated as + 1) capable of initiating translation (circRere), and the other containing a mutated start codon (ACC), which lacks translational initiation capability (δcircRere) (Fig. S1b, c). Both constructs were co-administered with a transfection reagent via intrathecal injection into the subarachnoid space of rats. Electrophysiological studies have revealed that compared to the spinal cord slices from the VCR group, both the frequency and amplitude of mEPSCs, as well as the enhanced neuronal firing of CaMKIIα-positive neurons in the VCR + circRere group were significantly ameliorated. However, there were no significant changes in the VCR + δcircRere group (Fig. 4a, b). Similarly, a down-right shift of the stimulus-response curve was evident in the VCR + circRere group compared with the VCR group, indicating that VCR-induced increasing of synaptic transmission mediated by afferent C-fibers is ameliorated (Fig. 4c). Subsequent behavioral tests showed that supplemental circRere through intrathecal injection effectively alleviated pain hypersensitivity in the VCR-treated rats (Fig. 4d-f). Intraspinal injection of recombinant AAV-hSyn-circRere, leading to the overexpression of circRere in neurons (Supplementary Fig. 5a), similarly attenuated the pain behavior (Fig. 4g-i). To further test whether the decrease of circRere in the dorsal horn neurons contributes to CINP, recombinant AAV-hSyn-circRere shRNA-EGFP was intraspinally injected into the L4-L6 spinal cord of naïve rats to knockdown of circRere. Twenty-one days after virus injection, there was a significant decrease in circRere expression, and a substantial co-labeling of green fluorescence with NeuN-positive cells, indicating high viral infection efficiency in neurons (Fig. S5b-d). Importantly, knockdown of the circRere resulted in the manifestation of pain behavior in rats (Fig. 4j-l). Taken together, these results collectively indicate that the decreased expression of circRere is a sufficient and necessary condition for mediating CINP.

### cRERE binds to ERK and modulates its phosphorylation

**Fig. 5:**
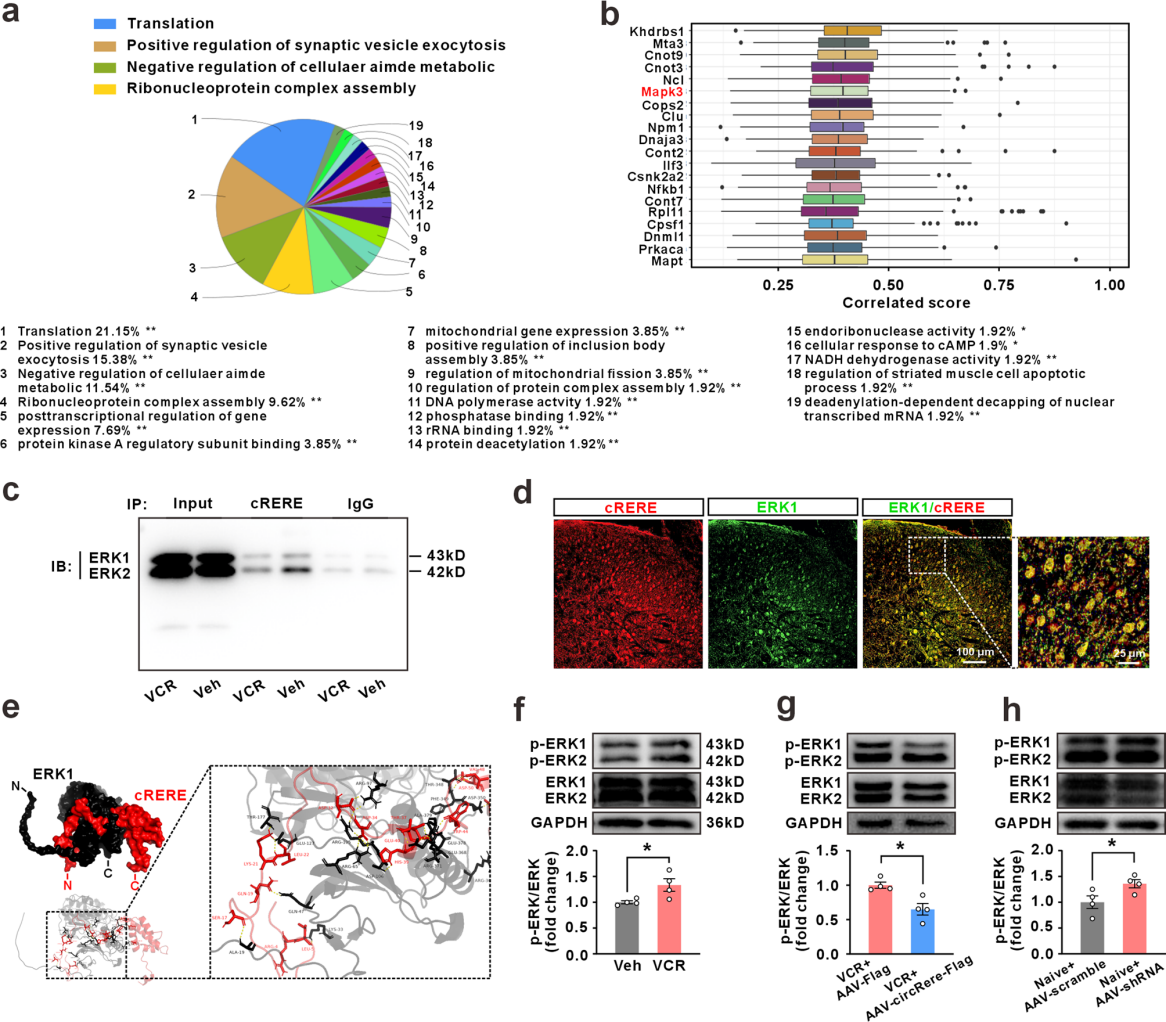
cRERE interacted with ERK1 and inhibits its phosphorylation. **a** The groups of terms after enrichment analysis of 208 potential cRERE-binding proteins. **b** Friend analysis of top 20 proteins among cRERE-binding proteins. **c** Co-IP validation of binding between cRERE and ERK1 in the spinal dorsal horn of naïve rats (*n* = 3 in each group). **d** The double-labelled immunofluorescence detection between cRERE and ERK1 in the Naïve rats (*n* = 3). **e** Molecular docking between cRERE and ERK1. The top left shows a 3D simulated structural diagram, the bottom left displays a simulated diagram containing protein secondary structure and 3D spatial information, while the magnified image on the right illustrates the main interaction sites between cRERE and ERK1. **f** The level of p-ERK1 was significantly increased in spinal dorsal horn of rats in 20 days after VCR treatment (* *P* < 0.05 vs. the vehicle group, Student’s t-test, *n* = 4 in each group). **g** The increased level of p-ERK1 in spinal dorsal horn induced by VCR was attenuated after overexpression of circRere via AAV virus (* *P* < 0.05 vs. the VCR + AAV-Flag group, Student’s t-test, *n* = 4 in each group). **h** The level of p-ERK1 in spinal dorsal horn of Naïve rats in 21 days after the intraspinal injection of AAV-scramble or AAV-shRNA (* *P* < 0.05 vs. the VCR + AAV-scramble group, Student’s t-test, *n* = 4 in each group). AAV-Flag represents AAV-hSyn-Flag, AAV-circRere-Flag represents AAV-hSyn-circRere-Flag, AAV-scramble represents AAV-hSyn-scramble-EGFP, AAV-shRNA represents AAV-hSyn-circRere shRNA-EGFP

To explore the downstream mechanism of how cRERE attenuates CINP, a co-immunoprecipitation coupled to LC-MS/MS detection was conducted in spinal dorsal horn tissue from naive rats. In total discovered 208 proteins might interact with the cRERE (Table S6). Our gene enrichment analysis using Cytoscape revealed that genes associated with regulating genome stability and protein synthesis accounted for more than 50% of all the identified results (Fig. 5a). Furthermore, Friends analysis of these 208 interacting proteins revealed that extracellular signal regulated kinase 1 (ERK1, also known as MAPK3, a classical molecule associated with pain [21–23]) had a strong correlation with the other genes (Fig. 5b). Co-immunoprecipitation confirmed that cRERE and ERK1 can indeed interact, and this interaction was reduced after treatment with VCR (Fig. 5c). High-resolution images also suggested that ERK1 was colocalized with the cRERE in spinal dorsal horn neurons (Fig. 5d). We also used the ClusPro server to perform molecular docking simulations of cRERE to the ERK1. The results showed that the unique N-terminal amino acid residues of cRERE interact with the amino acid residues in the middle segment helical structure of ERK1 through polar bonds (Fig. 5e). This spatial binding likely obstructs the phosphorylation sites (T202 and Y204) necessary for ERK1 activation. Western blotting results confirmed a significant upregulation of phosphorylated ERK1 (p-ERK1) rather than total ERK1 levels following VCR treatment (Fig. 5f). Intraspinal injection of AAV-hSyn-circRere attenuated the increased level of p-ERK1 induced by VCR (Fig. 5g). Inversely, intraspinal injection of AAV-hSyn-circRere shRNA-EGFP increased the expression of p-ERK1 in naïve rats (Fig. 5h). These results show that cRERE encoded by circRere inhibits the activation of ERK1 phosphorylation, it is reasonable to speculate that cRERE may involve in the process of CINP by influencing downstream signaling pathways regulated by kinase function.

### cRERE corrects vincristine-induced abnormal activation of the CREB/IL-1β signaling pathway via ERK modulation

**Fig. 6:**
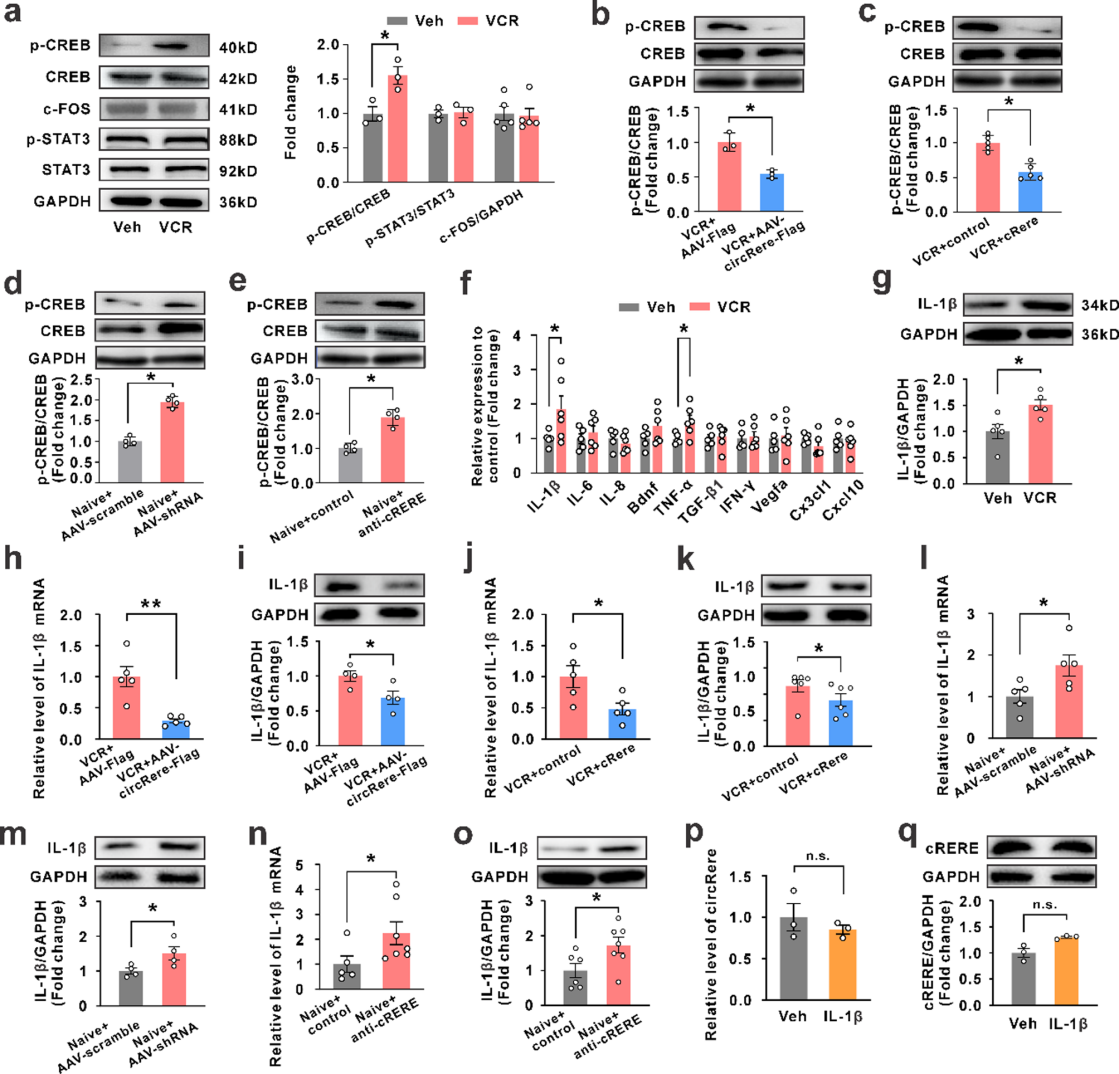
ERK1 activates p-CREB/IL-1β signaling pathway, contributing to CINP. a Left: The representative western blotting bands of p-CREB, CREB, c-FOS, p-STAT3, STAT3 and GAPDH protein in the spinal dorsal horn with treatment of vehicle or VCR. Right: The fold change of protein level of p-CREB/CREB, p-STAT3/STAT3 and c-FOS/GAPDH (*P < 0.05 vs. the vehicle group, Student’s t-test, *n* = 5 in each group). **b** The protein level of p-CREB/CREB in the spinal dorsal horn of VCR rats after intraspinal injection of AAV-Flag or AAV-circRere-Flag (* *P* < 0.05 vs. the VCR + AAV-Flag group, Student’s t-test, *n* = 5 for VCR + AAV-Flag group and *n* = 6 for VCR + AAV-circRere-Flag group) **c** The protein level of p-CREB/CREB in the spinal dorsal horn after intrathecal injection of cRERE or control (boiled cRERE) (* *P* < 0.05 vs. the VCR + control group, Student’s t-test, *n* = 5 in each group). **d** and **e** Compared to the corresponding control group, the protein expression of pCREB/CREB decreases in the spinal cord of naive rats after knocking down circRere or neutralizing cRERE (* *P* < 0.05 and ** *P* < 0.01 vs. the corresponding control group, Student’s t-test, *n* = 5 in each group for d and *n* = 4 n each group for e). **f** The mRNA level of ten cytokines in the spinal dorsal horn of vehicle or VCR-treated rats. (* *P* < 0.05 vs. the vehicle group, Student’s t-test, *n* = 5 for vehicle group and *n* = 6 for VCR group). **g** VCR but not vehicle treatment upregulated the protein level of IL-1β (* *P* < 0.05 vs. the vehicle group, Student’s t-test, *n* = 5 in each group). *h* and *i* Intraspinal injection of AAV-cicRere-Flag overexpressing circRere, significantly reduced VCR-induced increase in IL-1β mRNA and protein expression (* *P* < 0.05 and ** *P* < 0.01 vs. the VCR + AAV-Flag group, Student’s t-test,*n* = 5 in each group for h and *n* = 4 in each group for i). **j** and *k* Supplementing exogenous cRERE through intrathecal injection effectively alleviated the VCR-induced increase in IL-1β mRNA and protein expression (* *P* < 0.05 and ** *P* < 0.01 vs. the VCR + control group, Student’s t-test, *n* = 5 in each group for j and *n* = 6 in each group for k). **l** and **m** The mRNA and protein level of IL-1β in the spinal dorsal horn after intraspinal injection of AAV-scramble or AAV-circRere shRNA (* *P* < 0.05 vs. the Naïve + AAV-scramble group, Student’s t-test, *n* = 5 in each group for l and *n* = 4 n each group for m) **n** and *o* The mRNA and protein level of IL-1β in the spinal dorsal horn after intrathecal injection of control (boiled anti-cRERE) or anti-cRERE (* *P* < 0.05 vs. the Naïve + control, group, Student’s t-test, *n* = 5–7 in each group). **p** and **q** The expression level of circRere and cRERE in the spinal dorsal horn after intrathecal injection of IL-1β (n.s., no significance, Student’s t-test, *n* = 3 in each group). AAV-Flag represents AAV-hSyn-Flag, AAV-circRere-Flag represents AAV-hSyn-circRere-Flag, AAV-scramble represents AAV-hSyn-scramble-EGFP, AAV-shRNA represents AAV-hSyn-circRere shRNA-EGFP

Subsequently, we integrated results from literature and our previous work to identify three potential ERK1 downstream signaling pathways, namely cAMP-response element binding protein (CREB), immediate early gene (FOS), and signal transducer and activator of transcription 3 (STAT3), that may paly vital roles in pain modulatiuon [24–26]. The western blotting results indicated a significant upregulation of phosphorylated CREB (p-CREB) expression in the spinal dorsal horn of rats treated with VCR, while FOS and STAT3 did not show significant changes (Fig. 6a). Further, we found that both application of AAV-hSyn-circRere and cRERE could effectively reduce the upregulation of protein expression of p-CREB induced by VCR (Fig. 6b, c). Inversely, both application of AAV-hSyn-circRere shRNA-EGFP and anti-cRERE increased the p-CREB expression in naïve rats (Fig. 6d, e). These results confirmed that the expression changes of p-CREB were indeed regulated by circRere and its encoded cRERE. Given that p-CREB is a crucial regulatory intermediate in signaling pathways, involved in regulating the expression of various pain-related cytokines, we conducted network database searches and literature reviews to identify 10 cytokines potentially regulated by p-CREB and associated with pain [27–29], and their expression levels were assessed. QPCR results revealed upregulation of multiple cytokines post-VCR treatment, with IL-1β showed the most significant upregulation (Fig. 6f). The western blotting results also demonstrated a significant increase in IL-1β protein levels post-VCR treatment (Fig. 6g). Importantly, both application of AAV-hSyn-circRere and cRERE effectively reduced the increase protein and mRNA expression of IL-1β induced by VCR (Fig. 6h-k). Inversely, both application of AAV-hSyn-circRere shRNA-EGFP and anti-cRERE increased the protein and mRNA expression of IL-1β in naïve rats (Fig. 6l-o). Besides, to validate the reverse feedback of IL-1β on circRere or cRERE, we analyzed the level of circRere and cRERE after intrathecal injection of IL-1β. The results showed no change on the level of them (Fig. 6p, q). These results collectively demonstrate that the circRere encoded functional novel protein cRERE inhibits the activation of the p-CREB/IL-1β pathway by affecting ERK1 phosphorylation.

## Discussion

In the present study, we first identified the expression of circRere in the dorsal horn neurons of rat and further discovered that circRere can translate the protein-cRERE through an m6A-driven translation mechanism. Under physiological condition, cRERE binds to ERK1, inhibiting its phosphorylation. Following VCR treatment, the downregulation of circRere led to reduced translation of cRERE, thereby inhibiting the phosphorylation site of ERK1. This activation of the downstream p-CREB/IL-1β pathway subsequently induced central sensitization in the spinal cord, resulting in CINP. Furthermore, it was found that supplementing circRere or cRERE was an effective approach to alleviate CINP.

Studies have shown that circRNAs expression levels in the human and mouse brain are higher than in other organs [30]. Similarly, we found that circRere has relatively high expression levels in the spinal cord and brain of normal rats. This tissue-specific expression suggests that circRNAs may play a crucial role in neurological disorders and indicates the potential of circRNAs-based drugs for targeting the central nervous system. We and our colleagues have conducted research on the involvement of circRNAs in neuropathic pain mechanisms. The focus has been on how upregulated circRNAs modulate transcription and translation or act as miRNA sponges to promote pain progression [18, 19, 31–33]. However, research directly addressing the involvement of circRNAs in CINP mechanisms is extremely limited, and studies focusing on the function of downregulated circRNA are even more scarce. Our present study fills this gap by focusing on circRere, which has translation potential and is downregulated after VCR treatment. Supplementing circRere or the cRERE encoded by it demonstrated a good therapeutic effect in the rat CINP model. Furthermore, conservative sequence analysis of circRere revealed highly homologous circRNAs in the human circRNA database. In our subsequent experimental planning, we aim to detect the levels of circRere and cRERE in cerebrospinal fluid samples from clinical CINP patients and further validate the analgesic effects of circRere and cRERE in primates to provide a more solid preclinical research basis for the development of analgesic drugs.

Additionally, Peptide or protein-based therapeutics have gained increasing clinical recognition due to their high specificity and potent bioactivity. A prominent example is semaglutide, a glucagon-like peptide-1 receptor agonist approved for the treatment of type 2 diabetes and obesity, which has demonstrated remarkable therapeutic efficacy since its market introduction [34, 35]. Despite these advances, many exogenously designed peptides still face limitations, including immunogenicity, rapid degradation, and challenges in delivery. To overcome these obstacles, peptides or small proteins encoded by endogenous biomolecules-particularly translational products derived from non-coding RNAs with newly discovered coding potential-have emerged as attractive alternatives [36]. Among these, endogenous circular RNAs (circRNAs) are especially promising due to their covalently closed-loop structure, which confers superior stability, enhanced bioavailability, and reduced immunogenicity [7, 8]. Furthermore, products encoded by circRNAs exhibit evolutionary conservation and physiological relevance, enabling them to integrate more effectively into native cellular signaling pathways [37].

Currently known, circRNAs are primarily translated through four mechanisms: IRES, m6A modification, rolling circle amplification, and UTR translation activation elements [38, 39]. Some studies have reported that circRNAs mediated by IRES participate in disease progression [40, 41]. However, research on m6A-mediated circRNA translation initiation mechanisms are still scarce. Our present study revealed that circRere contains a clear cross-junction ORF, with the upstream sequence of the translation initiation site (within 100nt) including an m6A modification motif RRACH (R = G/A; H = A/C/U), strongly suggest that circRere can initiate translation and encode a protein. Furthermore, MeRIP and polysome profiling results confirmed that circRere in the spinal dorsal horn initiates translation through an m6A-dependent manner and produces the protein cRERE. Subsequently, through molecular docking simulations, we identified that a polar bond is formed between the 22nd amino acid residue of cRERE and the catalytic hinge region (amino acid residues 124–127) of ERK1, where ERK1 exhibits protein kinase activity. Our research, as well as that of our colleagues, has demonstrated conclusively that the activation of ERK1 is a critical factor in inducing central sensitization and neuropathic pain [22, 24, 42]. Additionally, Erk1 functions as a pivotal signaling molecule, triggering a cascade of downstream signaling pathway changes upon phosphorylation activation [22, 43]. These have been comprehensively described in the CINP model established in this study.

**Fig. 7:**
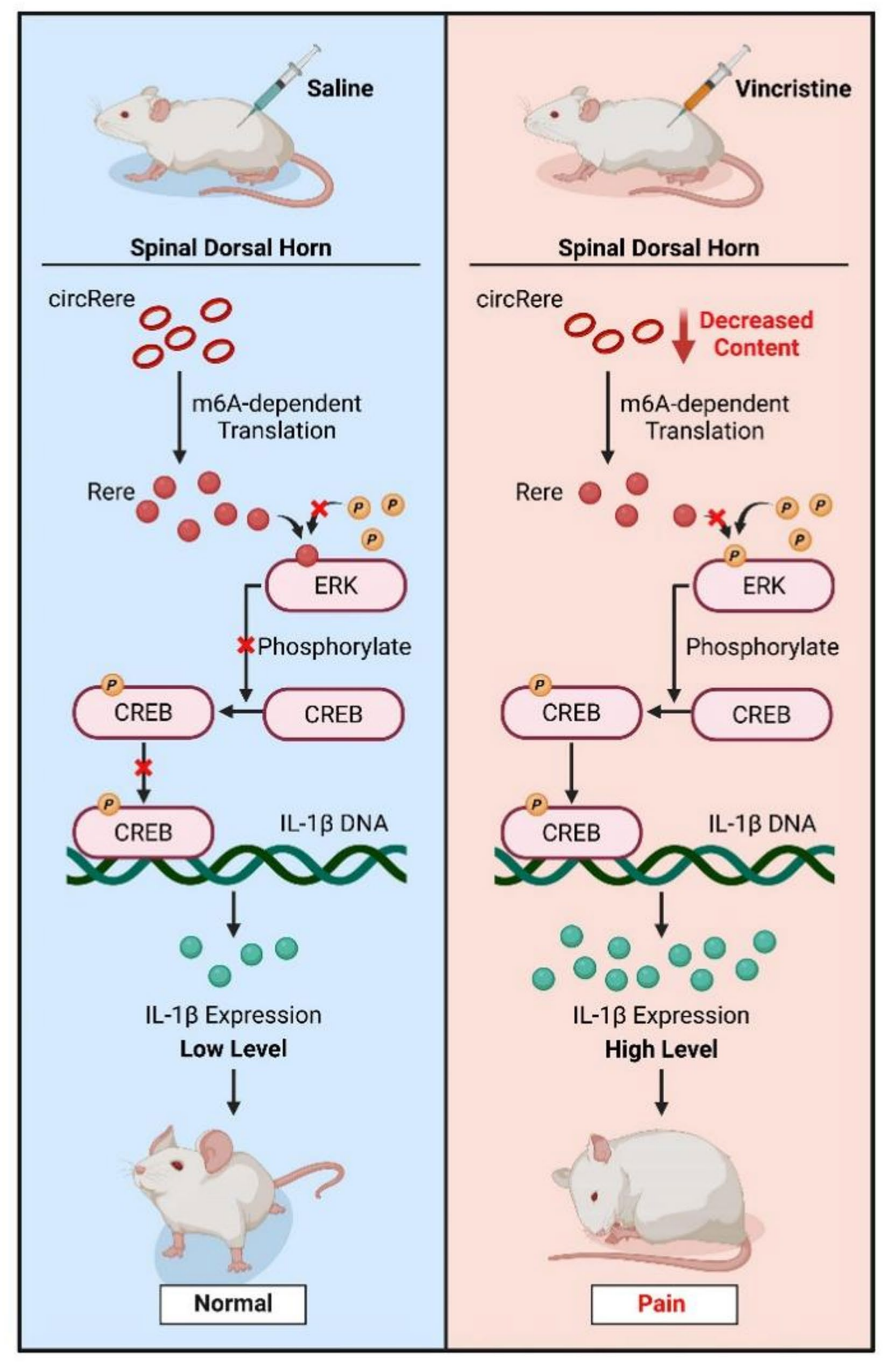
Summary graph for the hypothesis: the downregulation of cRERE encoded by circRere is involved in the mechanism of chemotherapy-induced neuropathic pain

In sammary, our results illustrated VCR treatment significantly downregulated circRere expression in the rat spinal cord dorsal horn, resulting in the reduction of the novel protein-cRERE it encoded. cRERE activated ERK1 in a disinhibitory manner, thereby activating the downstream CREB/IL-1β signaling pathway, inducing central sensitization and pain behavior. Our findings provide a reliable foundation for a more comprehensive understanding of the mechanisms of CINP and offer potential directions for clinically intervening in CINP in a rational manner (Fig. 7).

## Supplementary Information

Supplementary Material 1.

Supplementary Material 2.

Supplementary Material 3.

Supplementary Material 4.

Supplementary Material 5.

Supplementary Material 6.

Supplementary Material 7.

Supplementary Material 8.

The original format of Figure 1

The original format of Figure 2

## Data Availability

All the datasets presented in the paper are available from the corresponding author upon reasonable request. The circRNA sequencing data generated in this study have been deposited in Sequence Read Archive (SRA) under accession number PRJNA1328743.
