# Supplementary material for: A novel protein cRERE encoded by a circular RNA directly targets ERK signaling to alleviate chemotherapy-induced neuropathic pain
Source: Cell Commun Signal. 2025 Oct 17;23:445. doi: 10.1186/s12964-025-02455-x (PMC12535093; doi:10.1186/s12964-025-02455-x)
Supplement: Supplementary file 1 — Supplementary Material 1. [file 12964_2025_2455_MOESM1_ESM.docx]

**Supplementary Table 1. Specific primer sequences**

| **Gene name** | **Type** | **Sequence (5'→3')** |
| --- | --- | --- |
| circRere | Forward | GAGGATGAAGTGGAGGCTGAATA |
|  | Reverse | AAGTTTGGCCTGATGGCTAGG |
| circAcvr2a | Forward | TGTTACCCCGAAACCACCCT |
|  | Reverse | AGTATAGCACCTGAGTAGGAACAAG |
| circPtpn4 | Forward | CCTTAAAAATAGCTCACCTTCCAGT |
|  | Reverse | GATTGGAGTGATGTCAGGAGGAA |
| circJmjd1c | Forward | GTCTGGCTAGGGTCCTTCCT |
|  | Reverse | TGTCACTGCGGTAGAGTTCC |
| circClint1 | Forward | TGCTCGTTGTTTCCGCTGTT |
|  | Reverse | CCCAGAAGGTCCCCAAGGAT |
| circVar_7162 | Forward | AGTCAACGCCAACAGAAGAGC |
|  | Reverse | GTAGCCGTGGTCCATAAAGCC |
| circMagi2 | Forward | CGAAAACTGGTCCTGGGTGG |
|  | Reverse | ACGTGTTGAGATTGGCGGAG |
| circApoo | Forward | TGACTGGGGATTACGAGGGT |
|  | Reverse | GGGTGAAGTAACTGACTGTGCC |
| circGtf2i | Forward | CCCTCAGAAAAACGGTGGAGG |
|  | Reverse | ACGCCCTCTTTCGGTTCCAA |
| circVar_18941 | Forward | AGGCGCACACTTACCTTCAC |
|  | Reverse | TGGGCCAGTCTGTGGAAACA |
| circVar_20771 | Forward | AGCTCCAAGAGGAAAGCGGG |
|  | Reverse | CATACTCACGCTGCCCATTCA |
| circFam172a | Forward | TGAGCTCCTGGAAAGTGACTG |
|  | Reverse | ACAGATTCCTTACCTGAACAGCA |
| circVar_26568 | Forward | CAGTTCCCACTGGAGATGCC |
|  | Reverse | GGCACACCTCCCTTCGTTG |
| circUsp6nl | Forward | CTCTCCAAAAAGCAAGCCCCT |
|  | Reverse | TCCAAGTGGCTGAAAATGCTGA |
| circSdhaf2 | Forward | AGCGGGCTCTTTTGGTT |
|  | Reverse | ATGACTGGGATATTTACTACTGG |
| circVar_30822 | Forward | TTGTCTTTTTAGGGGTGACAGGC |
|  | Reverse | TGGAGAAAGAGGAGGGGATGTG |
| circVar_34279 | Forward | GTAGCCCTCTCTCCCTCTGTT |
|  | Reverse | TGGAATGAGGCTGCAAATGGTC |
| circVar_35860 | Forward | AAGATCACAGCTCCACCCCAG |
|  | Reverse | GAGGCATGTGATGGTGGCTA |
| circRhot1 | Forward | ACAAGCCGATGGATTCCTCTCA |
|  | Reverse | AACACATGAAGGTGGCATCGT |
| circVar_39042 | Forward | TGTTACACTCCCAGCCCATC |
|  | Reverse | TTTGCAGTAGGAAAGCTGCCG |
| circLrrc16b | Forward | ATGGGACATCCTGCACCTGA |
|  | Reverse | GTCCTGAGAACCAGCCAGGA |
| Rere | Forward | AAGCTGATCGAGAAGTGCTG |
|  | Reverse | CTCGAATTTCTCCTTGGGT |
| Mettl14 | Forward | AGGAACCTGAGATTGGCAACATAG |
|  | Reverse | TGTCCCTCCCGAAGAGATGAAG |
| ERK | Forward | AGCGGGCTGTCTCTTGGAAG |
|  | Reverse | AGATGAACCAGTGGCTGAGGAG |
| c-FOS | Forward | CAAACCGACCTACTGTCCC |
|  | Reverse | ACCAACAACCTTGTCGTCATAT |
| CREB | Forward | ACTCAGCCGGGTACTACCAT |
|  | Reverse | CATCACCAGAGGCAGCTTGA |
| STAT3 | Forward | GGAGGAGGCATTCGGAAAGT |
|  | Reverse | TCCAAACTGCATCAATGAATGGT |
| IL-1β | Forward | TCTCACAGCAGCATCTCGACAAG |
|  | Reverse | CCACGGGCAAGACATAGGTAGC |
| IL-6 | Forward | AGACTTCCAGCCAGTTGCCTTC |
|  | Reverse | GGTCTGTTGTGGGTGGTATCCTC |
| IL-8 | Forward | ACGCTGGCTTCTGACAACACTAGT |
|  | Reverse | CTTCTCTGTCCTGAGACGAGAAGG |
| Bdnf | Forward | GTGACARTATTAGCGAGTGGG |
|  | Reverse | GGGTAGTTCGGCATTGC |
| TNF-α | Forward | CCGAGATGTGGAACTGGCAGAG |
|  | Reverse | CCACGAGCAGGAATGAGAAGAGG |
| TGF-β1 | Forward | CTTGCCCTCTACAACCAACA |
|  | Reverse | CTTGCGACCCACGTAGTAGA |
| IFN-γ | Forward | GAGTTCATTGACAGGTTTGTGCTGG |
|  | Reverse | CTGAAGAAGTTAGTGATCAGGTGCG |
| Vegfa | Forward | CACGACAGAAGGGGAGCAGAAAG |
|  | Reverse | GGCACACAGGACGGCTTGAAG |
| Cx3cl1 | Forward | ACAAGATGACCTCGCCAATCCC |
|  | Reverse | GTGTCTGTGCTGTCTCGTCTCC |
| Cxcl10 | Forward | CGTGCTGCTGAGTCTGAGTGG |
|  | Reverse | AGCGGCTGTTCATGGAAGTCG |
| GAPDH | Forward | GTCAGTGCCGGCCTCGTCTCATA |
|  | Reverse | GACCCTTTTGGCACCACCCTTCA |
| 18S | Forward | ACACGGACAGGATTGACAGATTG |
|  | Reverse | GCCAGAGTCTCGTTCGTTATCG |
