# Supplementary material for: A novel protein cRERE encoded by a circular RNA directly targets ERK signaling to alleviate chemotherapy-induced neuropathic pain
Source: Cell Commun Signal. 2025 Oct 17;23:445. doi: 10.1186/s12964-025-02455-x (PMC12535093; doi:10.1186/s12964-025-02455-x)
Supplement: Supplementary file 2 — Supplementary Material 2. [file 12964_2025_2455_MOESM2_ESM.docx]

**Supplementary Table 2. Information of 21 significant decreased circRNAs**

| circName | gene symbol | Control-DAY20 | | Control-DAY10 | | counts | | |
| --- | --- | --- | --- | --- | --- | --- | --- | --- |
|  |  | log_2_FC | p-adj | log_2_FC | p-adj | control | DAY10 | DAY20 |
| rat_circ:chr3:33003598-33006797 | Acvr2a | -6.63E+00 | 1.33E-04 | -9.45E+00 | 4.28E-02 | 82 | 22 | 54 |
| rat_circ:chr20:22822066-22856689 | Jmjd1c | -6.99E+00 | 2.35E-06 | -9.81E+00 | 1.15E-02 | 97 | 46 | 96 |
| rat_circ:chr10:30980709-30993573 | Clint1 | -7.24E+00 | 7.97E-08 | -1.01E+01 | 4.27E-03 | 77 | 22 | 46 |
| rat_circ:chr5:167547693-167581074 | Rere | -6.61E+00 | 1.54E-04 | -9.44E+00 | 4.41E-02 | 73 | 35 | 0 |
| rat_circ:chr14:69800154-69819258 | * | -8.46E+00 | 1.36E-18 | -1.13E+01 | 3.52E-05 | 333 | 158 | 350 |
| rat_circ:chr4:11098246-11152065 | Magi2 | -6.79E+00 | 2.11E-05 | -9.60E+00 | 2.53E-02 | 251 | 183 | 250 |
| rat_circ:chrX:63552834-63602914 | Apoo | -6.58E+00 | 2.07E-04 | -9.41E+00 | 4.84E-02 | 18 | 16 | 16 |
| rat_circ:chr13:35769419-35784163 | Ptpn4 | -6.63E+00 | 1.33E-04 | -9.45E+00 | 4.28E-02 | 16 | 6 | 12 |
| rat_circ:chr12:25431710-25435591 | Gtf2i | -9.53E+00 | 2.86E-33 | -1.23E+01 | 2.71E-06 | 1850 | 727 | 1012 |
| rat_circ:chr8:115870141-115932067 | * | -9.37E+00 | 6.99E-31 | -1.22E+01 | 3.21E-06 | 546 | 198 | 554 |
| rat_circ:chr16:23687864-23729219 | * | -7.76E+00 | 1.04E-11 | -1.05E+01 | 6.66E-04 | 79 | 28 | 73 |
| rat_circ:chr2:4961753-5031767 | Fam172a | -7.77E+00 | 9.47E-12 | -1.05E+01 | 6.63E-04 | 133 | 78 | 125 |
| rat_circ:chr1:124576794-124584139 | * | -6.60E+00 | 1.79E-04 | -9.42E+00 | 4.62E-02 | 79 | 0 | 88 |
| rat_circ:chr17:75797811-75816925 | Usp6nl | -6.88E+00 | 8.42E-06 | -9.58E+00 | 2.76E-02 | 84 | 0 | 53 |
| rat_circ:chr1:226566338-226566744 | Sdhaf2 | -7.87E+00 | 1.27E-12 | -1.07E+01 | 3.26E-04 | 5 | 0 | 0 |
| rat_circ:chr9:94463647-94481167 | * | -1.15E+01 | 1.01E-69 | -1.44E+01 | 2.22E-05 | 6606 | 3677 | 6147 |
| rat_circ:chr5:76925720-76926914 | * | -9.39E+00 | 3.67E-31 | -1.22E+01 | 3.21E-06 | 552 | 292 | 125 |
| rat_circ:chr3:104816866-104818862 | * | -8.02E+00 | 6.27E-14 | -1.08E+01 | 1.77E-04 | 219 | 164 | 119 |
| rat_circ:chr10:67579952-67587851 | Rhot1 | -6.99E+00 | 2.35E-06 | -9.81E+00 | 1.15E-02 | 91 | 0 | 0 |
| rat_circ:chr14:43458941-43461320 | * | -7.07E+00 | 8.68E-07 | -9.89E+00 | 8.42E-03 | 101 | 68 | 99 |
| rat_circ:chr15:34178909-34179719 | Lrrc16b | -7.82E+00 | 3.37E-12 | -1.06E+01 | 4.02E-04 | 187 | 138 | 163 |

Note: FC: Folder change; p-adj: adjusted P value
