# Supplementary material for: A novel protein cRERE encoded by a circular RNA directly targets ERK signaling to alleviate chemotherapy-induced neuropathic pain
Source: Cell Commun Signal. 2025 Oct 17;23:445. doi: 10.1186/s12964-025-02455-x (PMC12535093; doi:10.1186/s12964-025-02455-x)
Supplement: Supplementary file 3 — Supplementary Material 3. [file 12964_2025_2455_MOESM3_ESM.docx]

**Supplementary Table 3. Information of 20 decreased circRNAs with ORF spanning the circular junction and corresponding peptide**

| **circRNA** | **Peptide** | **Start** | **End** | **Length(nt)** | **aa. counts** | **Wt.(kD)** |
| --- | --- | --- | --- | --- | --- | --- |
| circAcvr2a | Peptide01 | 270 | 635 | 366 | 122 | 13.99 |
| circPtpn4 | Peptide02 | 341 | 1186 | 846 | 282 | 32.29 |
| circClint1 | Peptide03 | 14 | 985 | 972 | 324 | 37.14 |
|  | Peptide04 | 951 | 1028 | 78 | 26 | 3.10 |
| circVar_7162 | Peptide05 | 153 | 200 | 48 | 16 | 1.91 |
|  | Peptide06 | 176 | 211 | 36 | 12 | 1.55 |
|  | Peptide07 | 28 | 279 | 252 | 84 | 9.51 |
| circRere | Peptide08 | 125 | 382 | 258 | 86 | 9.27 |
|  | Peptide09 | 366 | 755 | 390 | 130 | 14.28 |
| circMagi2 | Peptide10 | 30 | 1418 | 1389 | 463 | 50.88 |
| circApoo | Peptide11 | 190 | 972 | 783 | 261 | 30.02 |
| circGtf2i | Peptide12 | 169 | 276 | 108 | 36 | 4.29 |
|  | Peptide13 | 272 | 550 | 279 | 93 | 10.78 |
| circVar_18941 | Peptide14 | 274 | 348 | 75 | 25 | 2.88 |
|  | Peptide15 | 248 | 556 | 309 | 103 | 11.50 |
| circVar_20771 | Peptide16 | 258 | 341 | 84 | 28 | 3.09 |
|  | Peptide17 | 223 | 927 | 705 | 235 | 26.37 |
| circFam172a | Peptide18 | 78 | 542 | 465 | 155 | 18.22 |
| circVar_26568 | Peptide19 | 291 | 1214 | 924 | 308 | 35.21 |
| circUsp6nl | Peptide20 | 234 | 287 | 54 | 18 | 2.44 |
| circSdhaf2 | Peptide21 | 289 | 441 | 153 | 51 | 6.10 |
| circVar_30822 | Peptide22 | 101 | 565 | 465 | 155 | 17.14 |
| circVar_34279 | Peptide23 | 25 | 1215 | 1191 | 397 | 42.60 |
| circVar_35860 | Peptide24 | 122 | 2071 | 1950 | 650 | 71.28 |
|  | Peptide25 | 1593 | 2102 | 510 | 170 | 19.20 |
| circRhot1 | Peptide26 | 27 | 293 | 267 | 89 | 10.12 |
|  | Peptide27 | 268 | 585 | 318 | 106 | 11.96 |
| circVar_39042 | Peptide28 | 34 | 303 | 270 | 90 | 9.97 |
| circLrrc16b | Peptide29 | 18 | 425 | 408 | 136 | 14.93 |

P.S. Start: The site of start codon from the junction site. End: The site of stop codon from the junction site. Length(nt): The length of ORF region in circRNA. aa. count: The lenth of peptides. Wt. (Da): The predicted molecular weight of peptides.
