# Supplementary material for: A novel protein cRERE encoded by a circular RNA directly targets ERK signaling to alleviate chemotherapy-induced neuropathic pain
Source: Cell Commun Signal. 2025 Oct 17;23:445. doi: 10.1186/s12964-025-02455-x (PMC12535093; doi:10.1186/s12964-025-02455-x)
Supplement: Supplementary file 4 — Supplementary Material 4. [file 12964_2025_2455_MOESM4_ESM.docx]

**Supplementary Table 4. CircRere blasted against the human circRNAs in circBase.**

| **Score** | **QStart** | **QEnd** | **Qsize** | **Identity** | **circRNA** | **Strand** | **Start** | **End** | **Span** |
| --- | --- | --- | --- | --- | --- | --- | --- | --- | --- |
| 272 | 47 | 373 | 373 | 92.10% | hsa_circ_0009567 | + | 157411 | 189018 | 31608 |
| 272 | 47 | 373 | 373 | 92.10% | hsa_circ_0009570 | + | 156380 | 187987 | 31608 |
| 272 | 47 | 373 | 373 | 92.10% | hsa_circ_0009571 | + | 131539 | 163146 | 31608 |
| 272 | 47 | 373 | 373 | 92.10% | hsa_circ_0009575 | + | 6090 | 37697 | 31608 |
| 272 | 47 | 373 | 373 | 92.10% | hsa_circ_0009576 | + | 14192 | 45799 | 31608 |
| 272 | 47 | 373 | 373 | 92.10% | hsa_circ_0009577 | + | 42774 | 74381 | 31608 |

Note: Link: The details of each blast results. QStart: The start points of circRere in blast. QEnd: The end points of circRere in blast. Qsize: The length of circRere. Identity: The match rate to the whole sequence of circRere. circRNA: The name of target human circRNA. Start: The start points of human circRNA in blast. End: The end points of human circRNA in blast. Span: The length of human circRNA involved in blast.
