# Supplementary material for: A novel protein cRERE encoded by a circular RNA directly targets ERK signaling to alleviate chemotherapy-induced neuropathic pain
Source: Cell Commun Signal. 2025 Oct 17;23:445. doi: 10.1186/s12964-025-02455-x (PMC12535093; doi:10.1186/s12964-025-02455-x)
Supplement: Supplementary file 5 — Supplementary Material 5. [file 12964_2025_2455_MOESM5_ESM.docx]

**Supplementary Table 5 The titer test of the customized anti-cRERE antibody via ELISA**

| **The dilute strength of antibody** | **Absorbance** | |
| --- | --- | --- |
|  | **Replicate1** | **Replicate2** |
| 1:250 | 1.989 | 1.984 |
| 1:1000 | 1.907 | 2.070 |
| 1:4000 | 2.046 | 2.098 |
| 1:16000 | 1.916 | 1.969 |
| 1:64000 | 1.471 | 1.537 |
| 1:256000 | 0.864 | 0.915 |
| 1:1024000 | 0.339 | 0.361 |
| 1%BSA | 0.014 | 0.015 |
