# Supplementary material for: A novel protein cRERE encoded by a circular RNA directly targets ERK signaling to alleviate chemotherapy-induced neuropathic pain
Source: Cell Commun Signal. 2025 Oct 17;23:445. doi: 10.1186/s12964-025-02455-x (PMC12535093; doi:10.1186/s12964-025-02455-x)
Supplement: Supplementary file 6 — Supplementary Material 6. [file 12964_2025_2455_MOESM6_ESM.docx]

**Supplementary Table 6 The list of 208 proteins interact with cRERE**

| **Symbol** | **ENTREZID** | **LFQ.intensity.VVehIgG** | **LFQ.intensity.VVehXXW** | **Unique.peptides** |
| --- | --- | --- | --- | --- |
| Parp4 | 361046 | 0 | 3664400 | 15 |
| Prps1 | 29562 | 0 | 912200 | 13 |
| Herc2 | 308669 | 0 | 441740 | 13 |
| A0A0G2JY13 | 1E+08 | 0 | 4828600 | 2 |
| A0A0G2JYV2 | 366952 | 0 | 13554000 | 8 |
| Arhgef11 | 78966 | 0 | 1414300 | 8 |
| Clu | 24854 | 0 | 2218000 | 2 |
| Ilf3 | 84472 | 0 | 1745100 | 7 |
| Tacc2 | 309025 | 0 | 1424600 | 7 |
| Camsap3 | 689074 | 0 | 632790 | 8 |
| A0A0G2K652 | 316228 | 0 | 1393400 | 28 |
| Mrpl33 | 1E+08 | 0 | 3364500 | 3 |
| Agap1 | 316611 | 0 | 5034700 | 3 |
| A0A0G2K8L9 | 689025 | 0 | 3130900 | 4 |
| Gramd3 | 307288 | 0 | 4393700 | 9 |
| Myo1e | 25484 | 0 | 1044900 | 8 |
| Mrps24 | 498406 | 0 | 2351800 | 3 |
| Cp | 24268 | 0 | 2461100 | 2 |
| Nucb1 | 84595 | 0 | 936400 | 3 |
| Dcp2 | 291604 | 0 | 3003100 | 6 |
| Yars | 313047 | 0 | 1608900 | 11 |
| Mrps31 | 290850 | 0 | 2447000 | 3 |
| Tep1 | 64523 | 0 | 1253900 | 20 |
| Itpr1 | 81678 | 0 | 2572300 | 18 |
| Slc25a10 | 170943 | 0 | 1944800 | 3 |
| C5 | 362119 | 0 | 13208000 | 7 |
| Mrpl42 | 299743 | 0 | 3574800 | 2 |
| Ndufs4 | 499529 | 0 | 2706300 | 3 |
| A0A8I5ZKR8 | 291006 | 0 | 905880 | 3 |
| Aga | 290923 | 0 | 5486800 | 4 |
| Masp1 | 64023 | 0 | 24131000 | 3 |
| Afg3l2 | 307350 | 0 | 40589000 | 4 |
| Mrpl3 | 300974 | 0 | 5444100 | 7 |
| Dlg4 | 29495 | 0 | 2470000 | 4 |
| Cope | 290659 | 0 | 7184900 | 10 |
| Pam | 25508 | 0 | 577900 | 4 |
| Sec16a | 1E+08 | 0 | 1142600 | 3 |
| Rab5a | 64633 | 0 | 1552300 | 4 |
| Ctbp1 | 29382 | 0 | 5255700 | 6 |
| Nfkb1 | 81736 | 0 | 3427100 | 17 |
| Psmc6 | 289990 | 0 | 780010 | 2 |
| Txnl1 | 140922 | 0 | 890180 | 2 |
| A0A8I5ZWK0 | 497915 | 0 | 968080 | 6 |
| Fip1l1 | 289582 | 0 | 2164000 | 4 |
| Pcbp2 | 363005 | 0 | 1833800 | 3 |
| Rpsa | 29236 | 0 | 1815300 | 4 |
| Gramd1b | 300644 | 0 | 3183900 | 8 |
| A0A8I6A052 | 317423 | 0 | 661950 | 7 |
| Mrps7 | 113958 | 0 | 5856000 | 8 |
| Tle3 | 84424 | 0 | 2268200 | 4 |
| Ncl | 25135 | 0 | 1812600 | 5 |
| Fxr1 | 361927 | 0 | 1781600 | 3 |
| Map3k5 | 365057 | 0 | 12767000 | 45 |
| A1m | 252922 | 0 | 8516500 | 2 |
| A0A8I6A3F1 |  | 0 | 4270100 | 2 |
| Prkra | 311130 | 0 | 2033200 | 4 |
| Fyttd1 | 360726 | 0 | 1000400 | 2 |
| Kif1a | 363288 | 0 | 971980 | 7 |
| Ptprd | 313278 | 0 | 436690 | 8 |
| Slc25a1 | 29743 | 0 | 3947600 | 6 |
| Akap7 | 361458 | 0 | 7990000 | 3 |
| Cacna1b | 257648 | 0 | 1978800 | 9 |
| Aldh7a1 | 291450 | 0 | 719620 | 3 |
| Mrpl10 | 691075 | 0 | 2699900 | 3 |
| Ndufs2 | 289218 | 0 | 3992300 | 7 |
| Pwp1 | 362856 | 0 | 1050100 | 3 |
| Prkaca | 25636 | 0 | 9560600 | 4 |
| Sfxn5 | 261737 | 0 | 3061600 | 4 |
| L3mbtl3 | 309550 | 0 | 11025000 | 2 |
| Ndufs7 | 362837 | 0 | 7497600 | 2 |
| Ambra1 | 59319 | 0 | 565670 | 3 |
| Vps35 | 25479 | 0 | 1336600 | 6 |
| A0A8I6AJM0 | 364676 | 0 | 1614700 | 7 |
| Fscn1 | 683788 | 0 | 1177300 | 3 |
| Rufy3 | 360921 | 0 | 1321400 | 3 |
| Snrpd3 | 687711 | 0 | 2270700 | 2 |
| Hdac11 | 297453 | 0 | 1386700 | 2 |
| A0A8I6AMP1 | 291969 | 0 | 2341100 | 3 |
| A0A8I6AP88 | 115769 | 0 | 917200 | 5 |
| Dcp1a | 361109 | 0 | 5187600 | 10 |
| Cct7 | 297406 | 0 | 1605900 | 5 |
| Cacnb4 | 58942 | 0 | 2532000 | 5 |
| Myrf | 293736 | 0 | 7034300 | 11 |
| Mapt | 29477 | 0 | 1723000 | 3 |
| A0A8I6ASR7 | 308458 | 0 | 1301000 | 11 |
| A0A8I6ATM0 | 282824 | 0 | 2223300 | 3 |
| Prkacb | 293508 | 0 | 6744100 | 3 |
| A0A8I6AW60 | 307641 | 0 | 6213200 | 7 |
| Edil3 | 688400 | 0 | 1254800 | 2 |
| Ptprf | 360406 | 0 | 674810 | 6 |
| A0A8I6GBT6 | 686590 | 0 | 890310 | 2 |
| Aak1 | 500244 | 0 | 1444300 | 10 |
| Ddb1 | 64470 | 0 | 1288200 | 3 |
| Psmc5 | 81827 | 0 | 1116700 | 4 |
| Rpl22l2 | 361923 | 0 | 6235900 | 2 |
| Ilf2 | 310612 | 0 | 5946000 | 9 |
| Arhgef2 | 310635 | 0 | 648920 | 5 |
| Pbxip1 | 310644 | 0 | 377800 | 3 |
| Hsd17b10 | 63864 | 0 | 1635800 | 4 |
| A0A8J8YNR6 | 287433 | 0 | 2664800 | 3 |
| Dnm1l | 114114 | 0 | 664310 | 3 |
| Caprin1 | 362173 | 0 | 1278300 | 2 |
| Rqcd1 | 301513 | 0 | 2449400 | 10 |
| Rab3a | 25531 | 0 | 1443400 | 3 |
| Prpf19 | 246216 | 0 | 4740200 | 9 |
| Klc1 | 171041 | 0 | 2492100 | 8 |
| Usmg5 |  | 0 | 2372800 | 2 |
| Npm1 | 25498 | 0 | 3417400 | 3 |
| Acsl3 | 114024 | 0 | 647960 | 3 |
| Galk2 | 296117 | 0 | 10634000 | 8 |
| Mrps9 | 301371 | 0 | 1406700 | 10 |
| Cnot11 | 363221 | 0 | 619480 | 3 |
| Sdhaf1 | 499125 | 0 | 2072300 | 2 |
| Erh | 681415 | 0 | 1976500 | 3 |
| Mrpl18 | 292244 | 0 | 4519400 | 4 |
| Arpc4 | 297518 | 0 | 3281600 | 3 |
| Cnot7 | 306492 | 0 | 1949000 | 6 |
| C1r | 312705 | 0 | 10786000 | 3 |
| Lgi2 | 305417 | 0 | 1126600 | 4 |
| Rbbp4 | 313048 | 0 | 7903200 | 3 |
| Ddx6 | 500988 | 0 | 1652700 | 9 |
| Zfp787 | 365176 | 0 | 1377200 | 4 |
| Fuk | 307848 | 0 | 673260 | 2 |
| Tnks1bp1 | 295707 | 0 | 3080800 | 23 |
| Ptcd3 | 500199 | 0 | 2992100 | 6 |
| Mrps23 | 360594 | 0 | 4785300 | 9 |
| Endod1 | 363015 | 0 | 836630 | 2 |
| Mrpl28 | 497876 | 0 | 6341500 | 8 |
| Trim2 | 361970 | 0 | 2999300 | 5 |
| Mtx3 | 688905 | 0 | 1567600 | 3 |
| Mrpl51 | 297601 | 0 | 2672100 | 2 |
| Mta3 | 1E+08 | 0 | 1527800 | 3 |
| Pnpla6 | 360753 | 0 | 429750 | 3 |
| Ndufa2 | 291660 | 0 | 2949200 | 4 |
| Map6d1 | 363823 | 0 | 809350 | 2 |
| Exog | 301062 | 0 | 1570400 | 6 |
| Cnot3 | 308311 | 0 | 776280 | 3 |
| Armcx4 | 1E+08 | 0 | 1357200 | 7 |
| Kifap3 | 289168 | 0 | 1715000 | 6 |
| Mrps11 | 499185 | 0 | 2223300 | 2 |
| Cep170b | 500726 | 0 | 841050 | 5 |
| Diras1 | 366826 | 0 | 927500 | 2 |
| Ndufa6 | 315167 | 0 | 8022700 | 2 |
| Mrpl19 | 297372 | 0 | 2312400 | 10 |
| Mrps16 | 688912 | 0 | 5793000 | 3 |
| Mrps35 | 297727 | 0 | 4584700 | 6 |
| Fam120a | 291019 | 0 | 889880 | 6 |
| Mgst3 | 289197 | 0 | 4284700 | 3 |
| Mrpl32 | 291206 | 0 | 3917600 | 2 |
| Dclk2 | 310698 | 0 | 1524500 | 9 |
| Fv1 | 308568 | 0 | 2193800 | 3 |
| Ppef1 | 317498 | 0 | 9427100 | 10 |
| Dpysl4 | 25417 | 0 | 531940 | 2 |
| Akap5 | 171026 | 0 | 2698200 | 10 |
| Hcn2 | 114244 | 0 | 38513000 | 1 |
| Ndufa13 | 1.01E+08 | 0 | 2852800 | 4 |
| Mrps6 | 1E+08 | 0 | 3992700 | 2 |
| Wwc1 | 303039 | 0 | 3112100 | 2 |
| F7F888 | 361724 | 0 | 2235900 | 8 |
| Coro1c | 501841 | 0 | 1616100 | 6 |
| Ppp1r18 | 361790 | 0 | 13551000 | 22 |
| Llgl1 | 54265 | 0 | 906870 | 5 |
| Dnaja3 | 360481 | 0 | 1656200 | 3 |
| Syt2 | 24805 | 0 | 2376200 | 3 |
| Elavl2 | 286973 | 0 | 4177700 | 2 |
| Atp6v1e1 | 297566 | 0 | 1750700 | 5 |
| Cnot1 | 291841 | 0 | 4734000 | 45 |
| LOC100911313 | 311328 | 0 | 1353600 | 4 |
| Cdc5l | 85434 | 0 | 1848700 | 2 |
| Rplp2 | 140662 | 0 | 12252000 | 5 |
| Rab2a | 65158 | 0 | 1095700 | 2 |
| Cltb | 116561 | 0 | 2981500 | 2 |
| Kras | 24605 | 0 | 3947400 | 3 |
| Gnaz | 25740 | 0 | 1401200 | 2 |
| Mapk3 | 50689 | 0 | 2117800 | 4 |
| Sdhb | 298596 | 0 | 2114400 | 3 |
| Pfkl | 25741 | 0 | 755900 | 2 |
| Aars |  | 0 | 1154400 | 5 |
| Ap3m2 | 171126 | 0 | 553850 | 2 |
| Cops2 | 261736 | 0 | 4279000 | 4 |
| Rpl11 | 362631 | 0 | 3335400 | 4 |
| Cdk5 | 140908 | 0 | 4128600 | 3 |
| LOC100911034 | 84594 | 0 | 1677200 | 4 |
| Ddhd1 | 305816 | 0 | 419710 | 3 |
| Mrpl2 | 301240 | 0 | 4654000 | 6 |
| Acsf2 | 619561 | 0 | 601990 | 3 |
| Copg1 | 297428 | 0 | 5289600 | 19 |
| Mrpl44 | 301552 | 0 | 4496700 | 4 |
| Hdac1 | 297893 | 0 | 1787700 | 2 |
| Mrps25 | 297459 | 0 | 1733300 | 5 |
| Nono | 317259 | 0 | 14359000 | 13 |
| Dnaja2 | 84026 | 0 | 1575200 | 4 |
| Cnot2 | 299805 | 0 | 1337900 | 7 |
| Rpl13a | 317646 | 0 | 12285000 | 5 |
| Steap3 | 170824 | 0 | 36920000 | 20 |
| Mrpl13 | 299938 | 0 | 3123400 | 2 |
| Gadd45gip1 | 288916 | 0 | 21039000 | 2 |
| Ehd1 | 293692 | 0 | 1135400 | 3 |
| Arcn1 | 300674 | 0 | 2552400 | 13 |
| Lancl2 | 362375 | 0 | 814570 | 2 |
| Slc27a1 | 94172 | 0 | 2269600 | 7 |
| Gnl1 | 309593 | 0 | 4099400 | 5 |
| C4a | 24233 | 0 | 138690000 | 3 |
| Ndufv3 | 64539 | 0 | 1808700 | 3 |
| Slc4a8 | 315311 | 0 | 972900 | 2 |
| Vtn | 29169 | 0 | 4737100 | 3 |
| Khdrbs1 | 117268 | 0 | 49551000 | 10 |
| Sacm1l | 116482 | 0 | 508550 | 3 |
