# Supplementary material for: A novel protein cRERE encoded by a circular RNA directly targets ERK signaling to alleviate chemotherapy-induced neuropathic pain
Source: Cell Commun Signal. 2025 Oct 17;23:445. doi: 10.1186/s12964-025-02455-x (PMC12535093; doi:10.1186/s12964-025-02455-x)
Supplement: Supplementary file 7 — Supplementary Material 7. [file 12964_2025_2455_MOESM7_ESM.docx]

**Supplementary Figures**

**
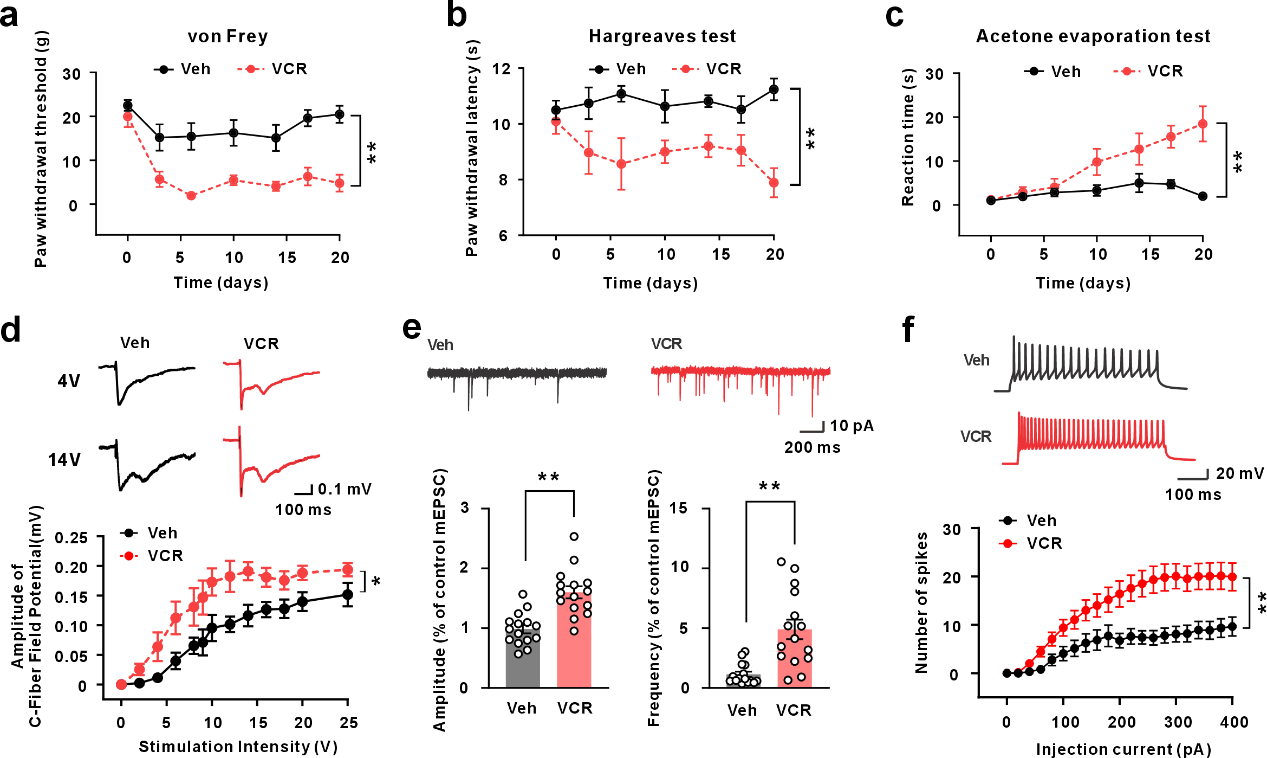
**

***Supplementary Figure 1.*** ***a*** *von Frey test of vincristine-treatment and vehicle rats (** P < 0.01 vs the vehicle group, one-way repeated-measures ANOVA, n = 10 in each group).* ***b*** *Hargreaves test of Vincristine-treatment and vehicle rats (** P < 0.01 vs the vehicle group, one-way repeated-measures ANOVA, n = 9 in each group).* ***c*** *Acetone evaporation test of Vincristine-treatment and vehicle rats (** P < 0.01 vs the vehicle group, one-way repeated-measures ANOVA, n = 10 in each group).* ***d*** *The stimulation-respond curve of C-fiber-evoked field potential in the spinal dorsal horn. The left charts were the representative wave form stimulated by 4 V and 14 V in vehicle or VCR group (* P < 0.05 vs the vehicle group, one-way repeated-measures ANOVA, n = 5 for vehicle group and n = 7 for VCR group).* ***e*** *The amplitude and frequency of mEPSCs increased significantly in the spinal dorsal horn of VCR rats (** P < 0.01 vs the vehicle group, Student’s t-test, n = 15 cells from 3 rats in each group).* ***f*** *The number of depolarization-induced neuronal firings increased significantly in the spinal dorsal horn of VCR rats (** P < 0.01 vs the vehicle group, Student’s t-test, n = 15 cells from 3 rats in each group).*


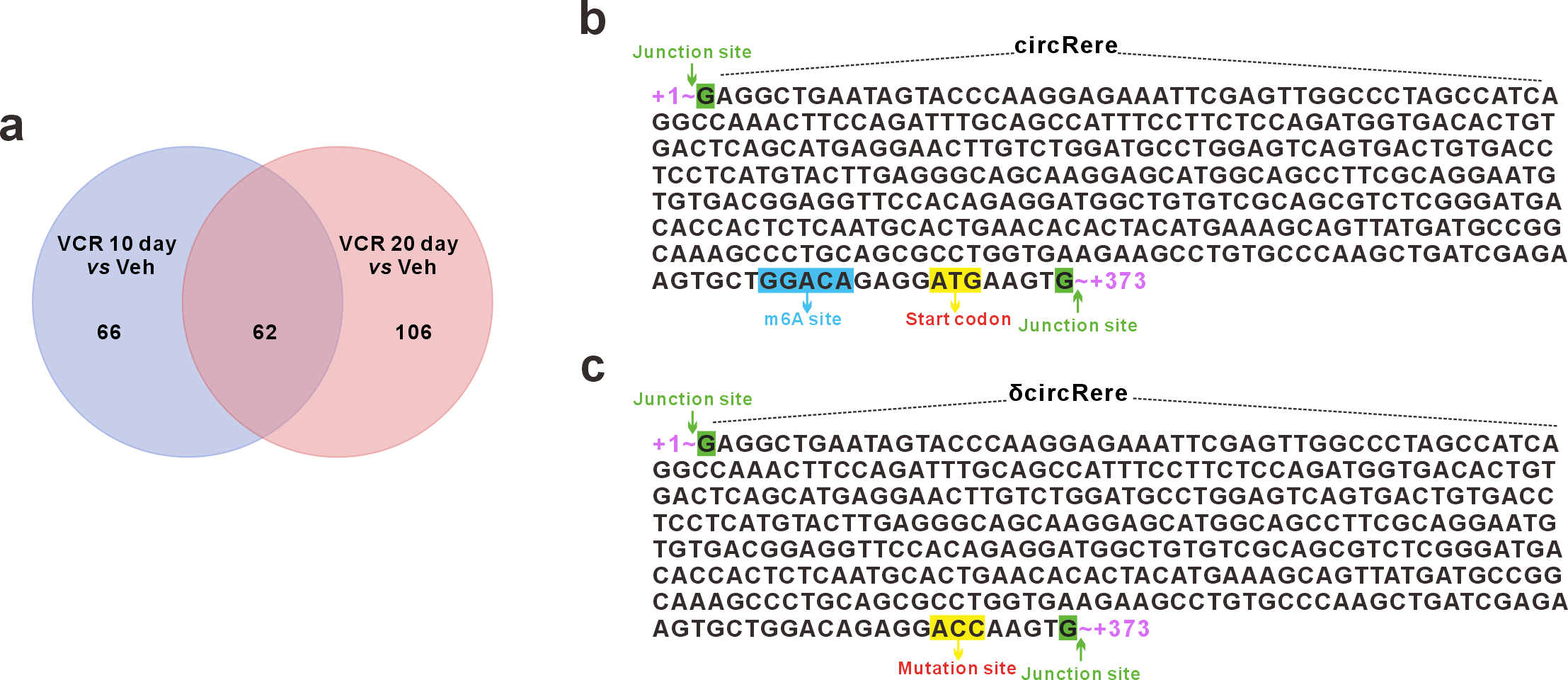


***Supplementary Figure 2.*** ***a*** *Compared with the control group, sixty-two* *common DEcRs were identified after 10 and 20 days after VCR treatment.* ***b*** *The total view of sanger sequencing of circRere and the total length of circRere was 373 bp (first nucleotide on the right side of the junction site designated as +1). The junction sites were marked as green base-GG and the start condon was marked as yellow base-ATG. The distance between two junction sites was 373 bp. C The sequence of δcircRere which has mutated the start codon. The junction sites were marked as green base-GG and the mutation site of start condon was marked as yellow base-ACC.*


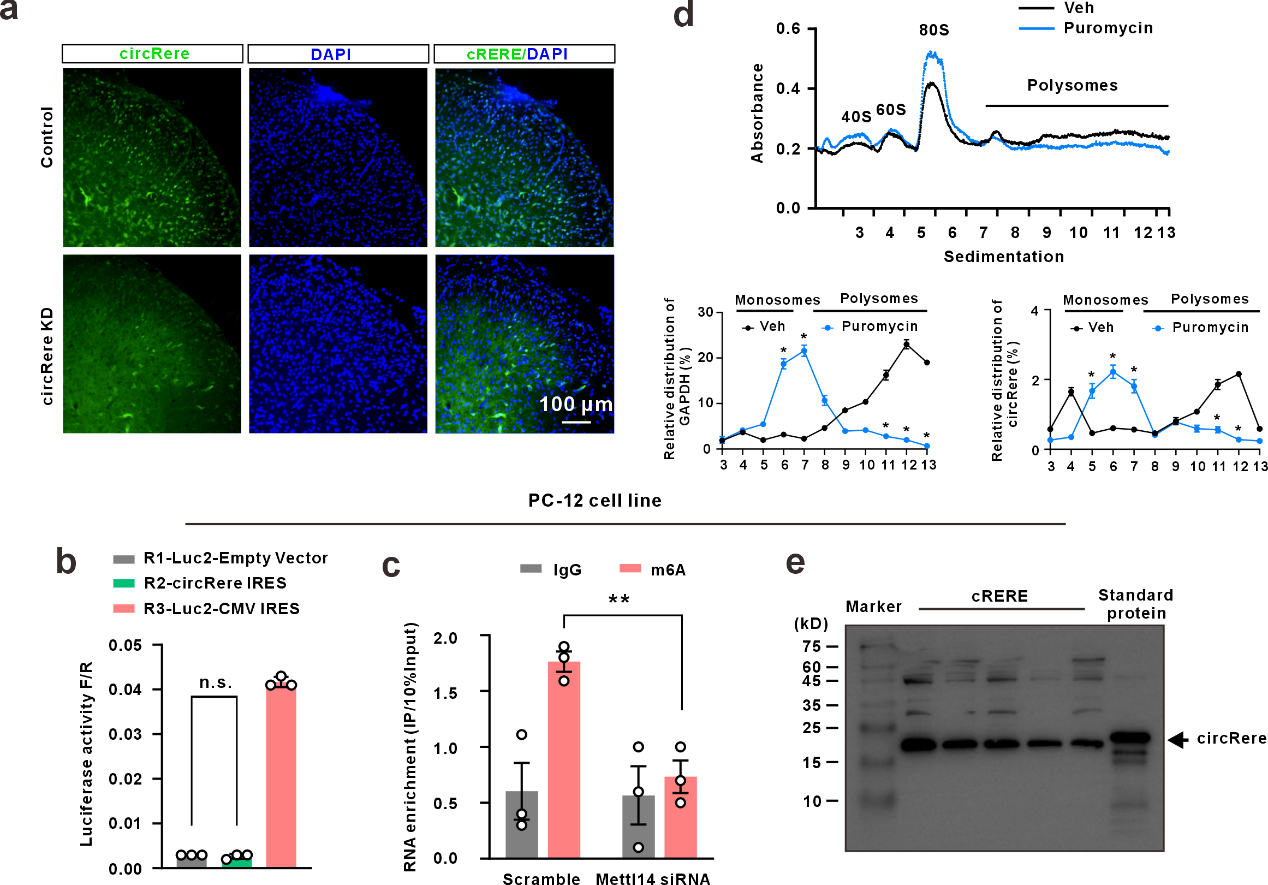


***Supplementary Figure 3. A*** *Validation of the specificity of the circRere probe.* ***b*** *Dual-luciferase assay to detect the IRES activity. R1-Luc2-Empty Vector was the negative control without any element driving translation. R3-Luc2-EMCV IRES was the positive control with a known element effectively driving translation. R2-Luc2-circRere IRES containing the IRES from circRere, which failed to initiate translation of luciferase (n.s., no significance, one-way ANOVA, n = 3 in each group).* ***c*** *MeRIP detection after treatment with scramble or Mettl14 siRNA, showing disruption m6A modification of circRere (* P < 0.05 vs the scramble group, Student’s t-test, n = 3 in each group).* ***d*** *Polysome profiling analysis of GAPDH and circRere (* P < 0.05 vs the vehicle group,* *Student’s t-test, n = 3 for each group).* ***e*** *The cRERE detected in PC-12 cells with a custom polyclonal antibody.*


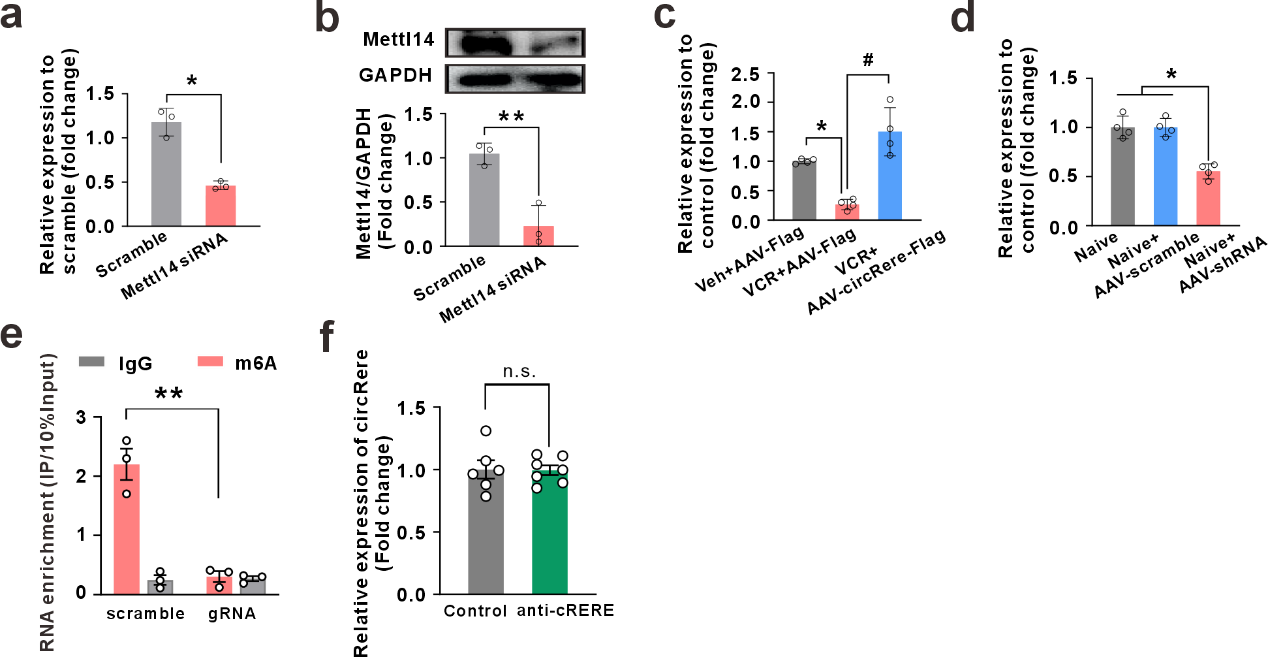


***Supplementary Figure 4. A and b*** *qPCR and western blotting analysis confirming decreased METTL14 mRNA and protein levels after siRNA treatment (* P < 0.05 vs the scramble group, Student’s t-test, n = 3 in each group).* ***c*** *The qPCR detection of spinal dorsal horn from rats with intraspinal injection of AAV-Flag and AAV-circRere-Flag (* P < 0.05 vs the veh+AAV-Flag group, # P < 0.05 vs the VCR+ AAV-Flag group, one-way ANOVA, n=4 in each group).* ***d*** *TheqPCR assay of spinal dorsal horn from rats with intraspinal injection of AAV-scramble and AAV-shRNA (* P < 0.05 vs the Naïve or Naive+AAV-scramble group, one-way ANOVA, n=4 in each group, AAV-).* ***e*** *Analysis of m6A levels in the circRere on day 5 after co-injection of CRISPR-dCasRx-Fto and gRNA into L4-L6 spinal dorsal horn in naive rats (* P < 0.05 vs the scramble group, Student’s t-test, n = 3 in each group).* ***f*** *The expression of circRere after intrathecal injection of control (boiled anti-cRERE) or anti-cRERE antibody (n.s., no significance, Student’s t-test, n = 6 for control group and n = 7 for anti-cRERE group). AAV-Flag represents AAV-hSyn-Flag, AAV-circRere-Flag represents AAV-hSyn-circRere-Flag, AAV-scramble represents AAV-hSyn-scramble-EGFP, AAV-shRNA represents AAV-hSyn-circRere shRNA-EGFP.*


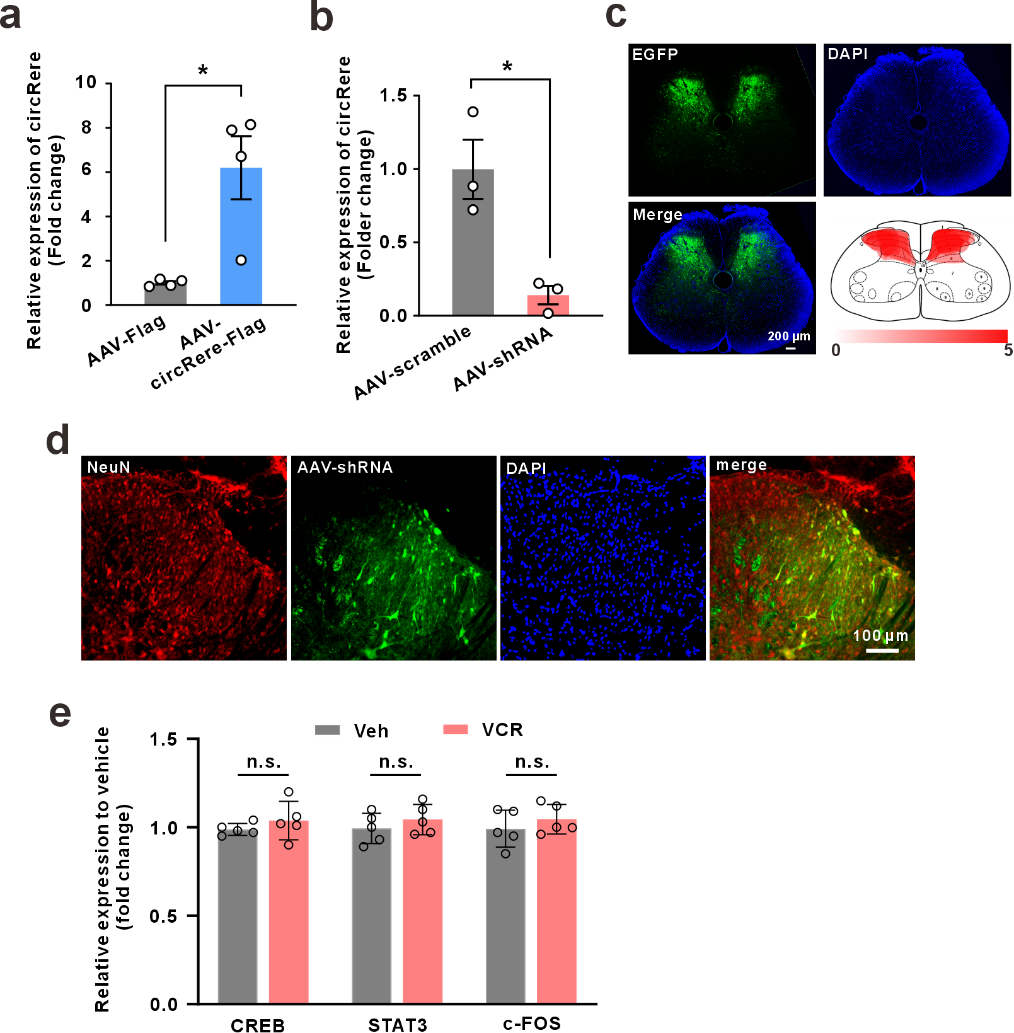


***Supplementary Figure 5.*** ***a*** *The qPCR detection of circRere on 21 days after intraspinal injection of recombinant AAV-Flag or AAV-circRere-Flag (* P < 0.05 vs the AAV-Flag group, Student’s t-test, n = 4 in each group).* ***b*** *The qPCR detection of circRere on 21 days after intraspinal injection of recombinant AAV-scramble or AAV-shRNA (* P < 0.05 vs the scramble-EGFP group, Student’s t-test, n = 3 in each group).* ***c*** *The presence of marked green fluorescence from AAV-shRNA on 21 days after virus injection.* ***d*** *The NeuN-positive cells colocalized with the marked green fluorescence of AAV-shRNA.* ***e*** *The mRNA expression level of several key molecules in the ERK signaling pathway (n.s., no significance, Student’s t-test, n = 5 in each group). AAV-Flag represents AAV-hSyn-Flag, AAV-circRere-Flag represents AAV-hSyn-circRere-Flag, AAV-scramble represents AAV-hSyn-scramble-EGFP, AAV-shRNA represents AAV-hSyn-circRere shRNA-EGFP.*
