# Supplementary material for: A novel protein cRERE encoded by a circular RNA directly targets ERK signaling to alleviate chemotherapy-induced neuropathic pain
Source: Cell Commun Signal. 2025 Oct 17;23:445. doi: 10.1186/s12964-025-02455-x (PMC12535093; doi:10.1186/s12964-025-02455-x)

Figure 1g


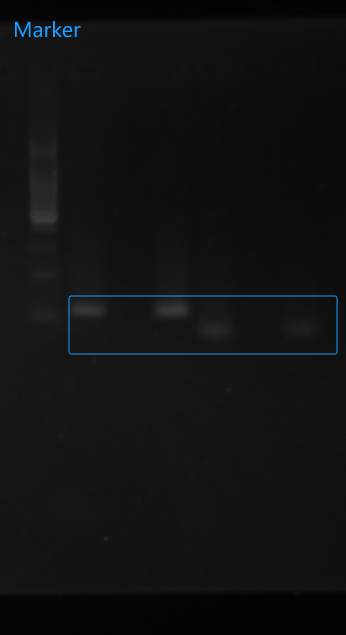


Figure 2f


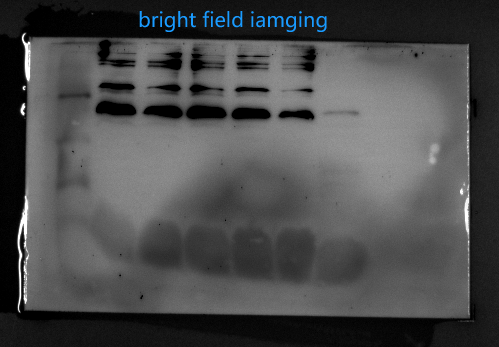


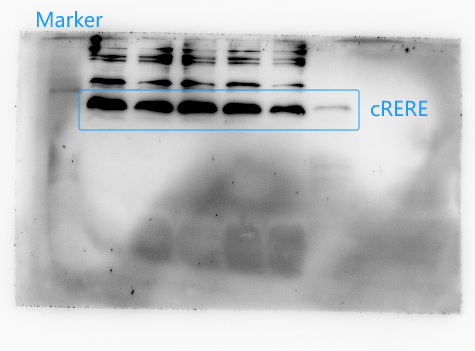


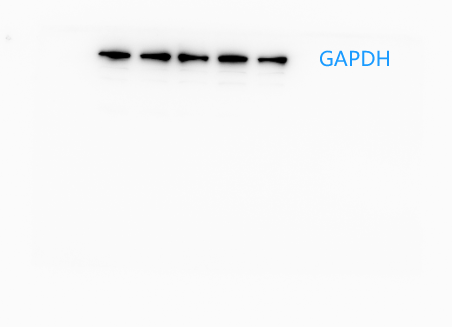


Figure 2i


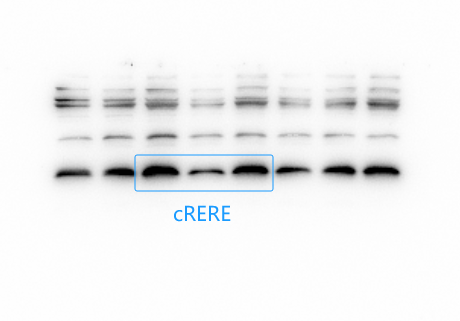


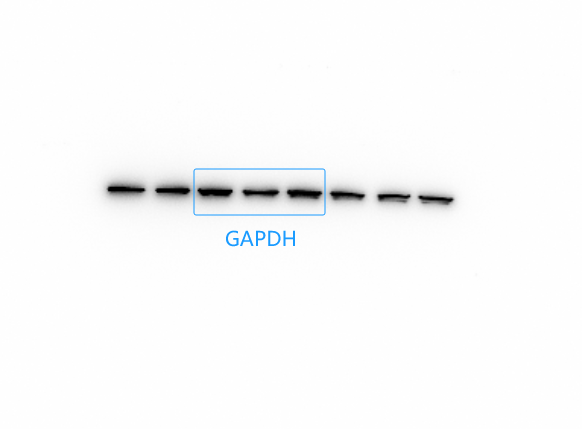


Figure 2j


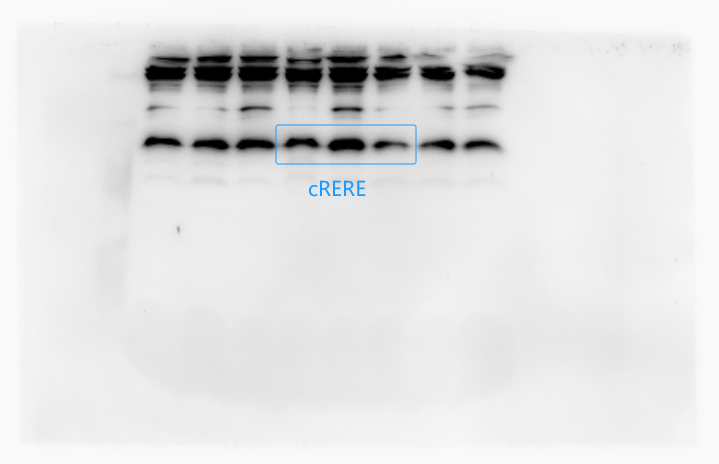


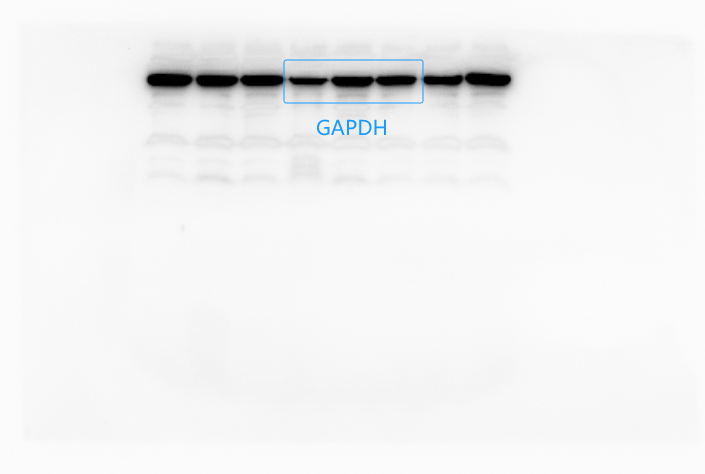


Figure 2l


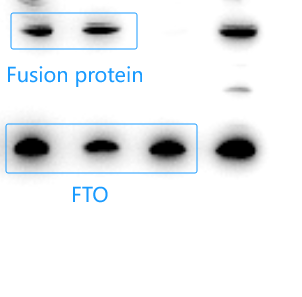


Figure 2m


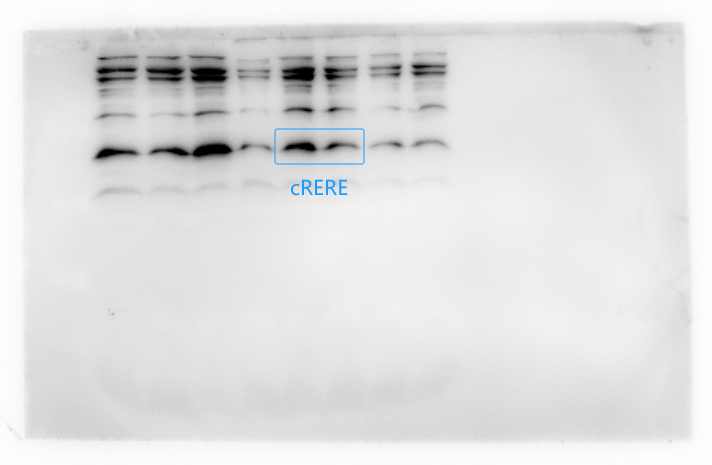


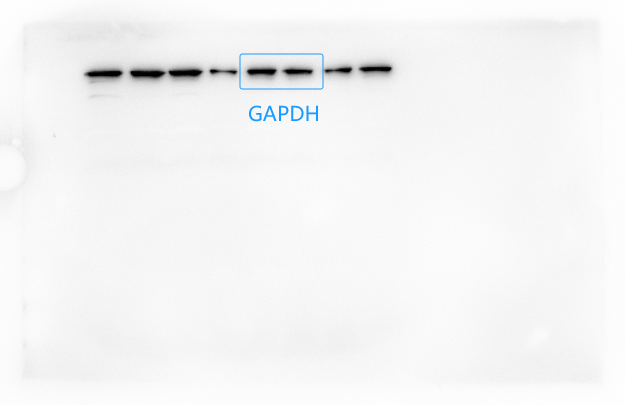


Figure 3a


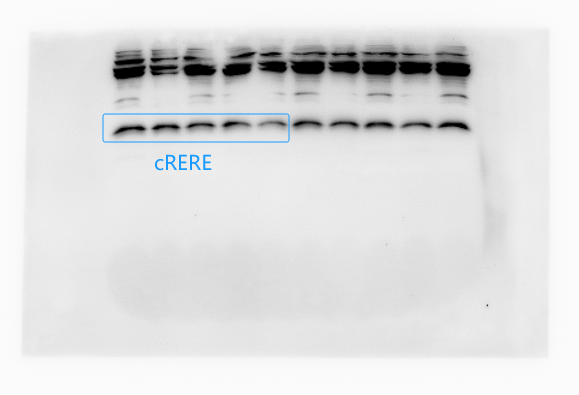


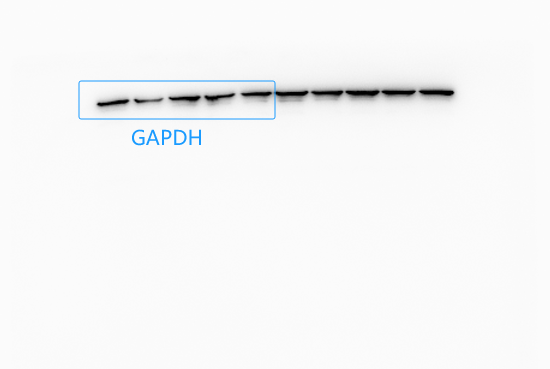


Figure 5c


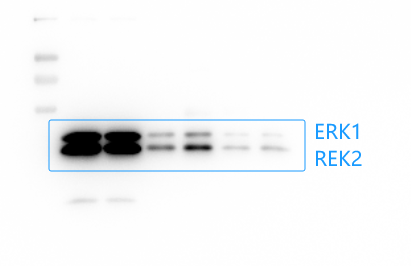


Figure 5f


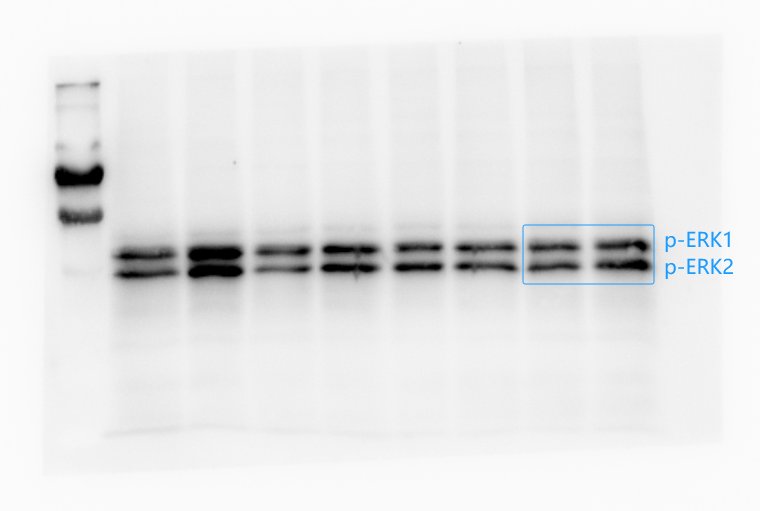


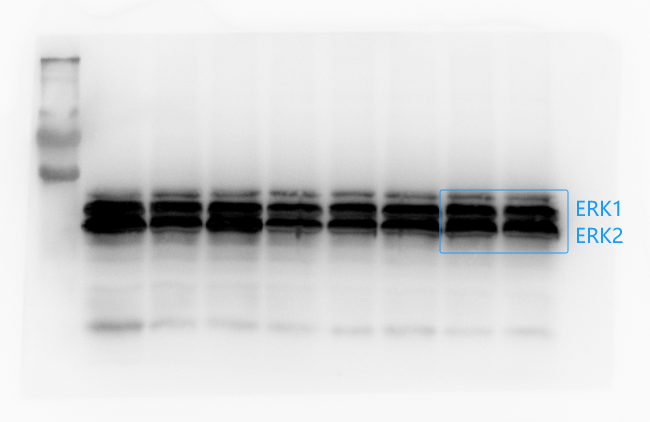


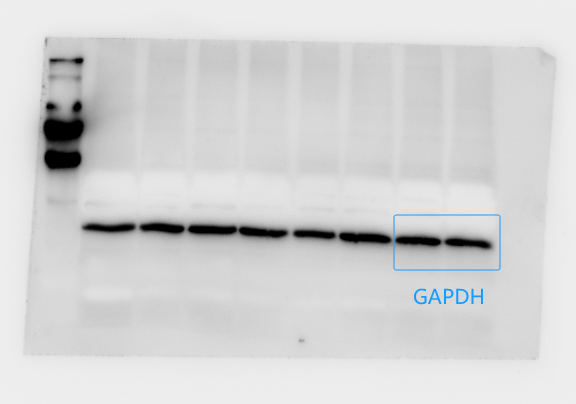


Figure 5g


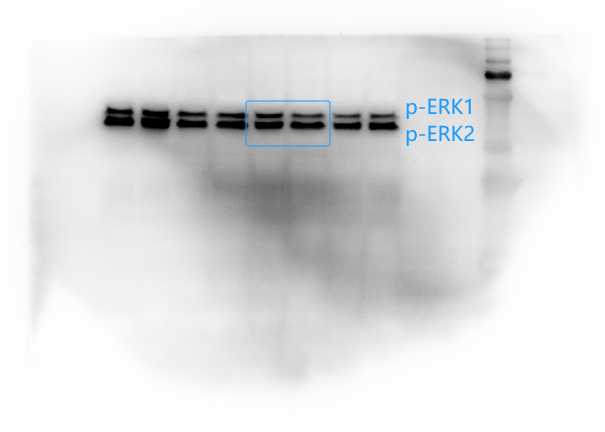


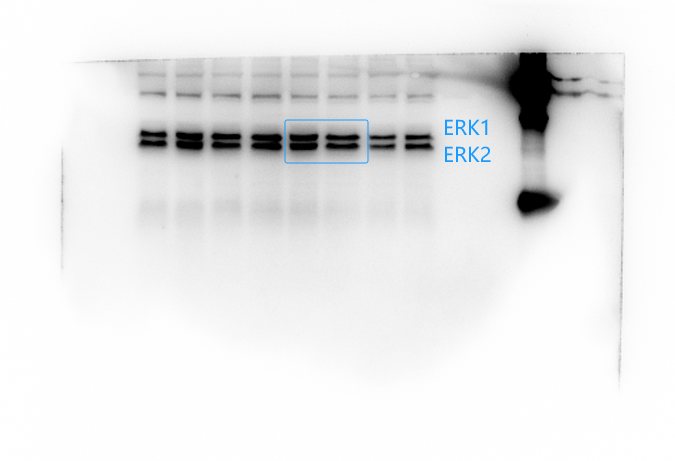


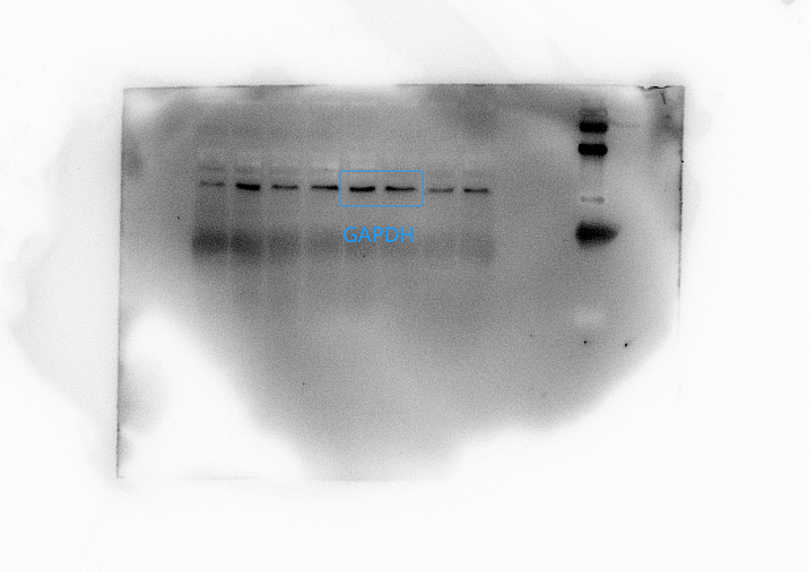


Figure 5h


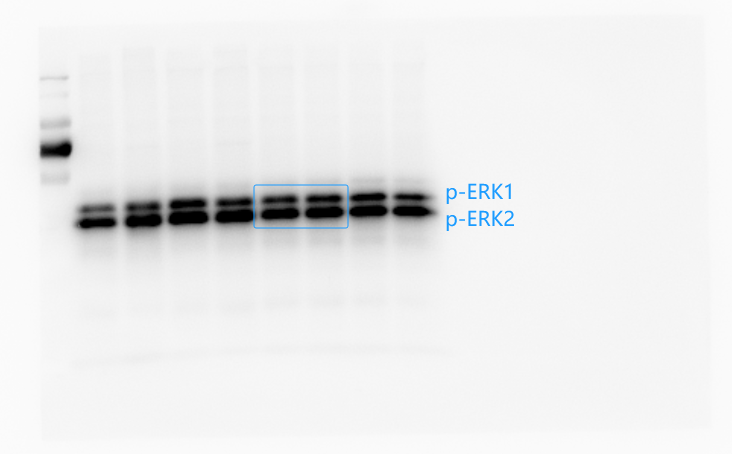


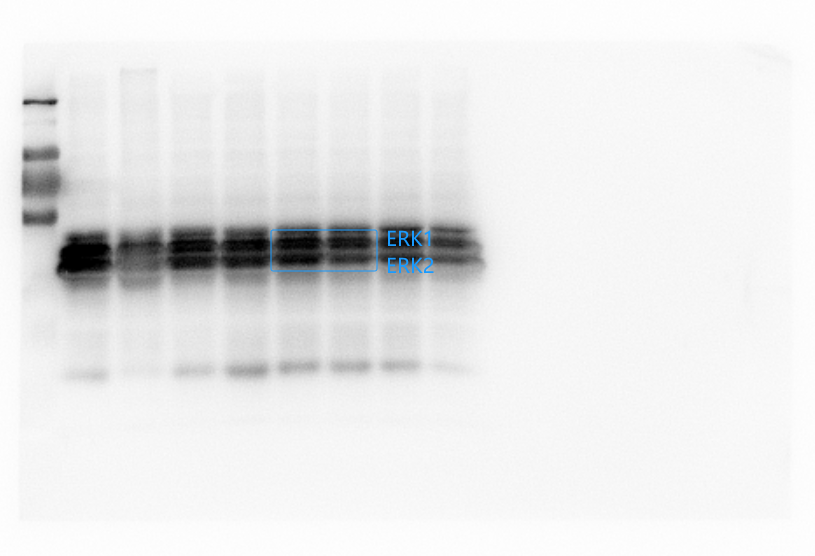


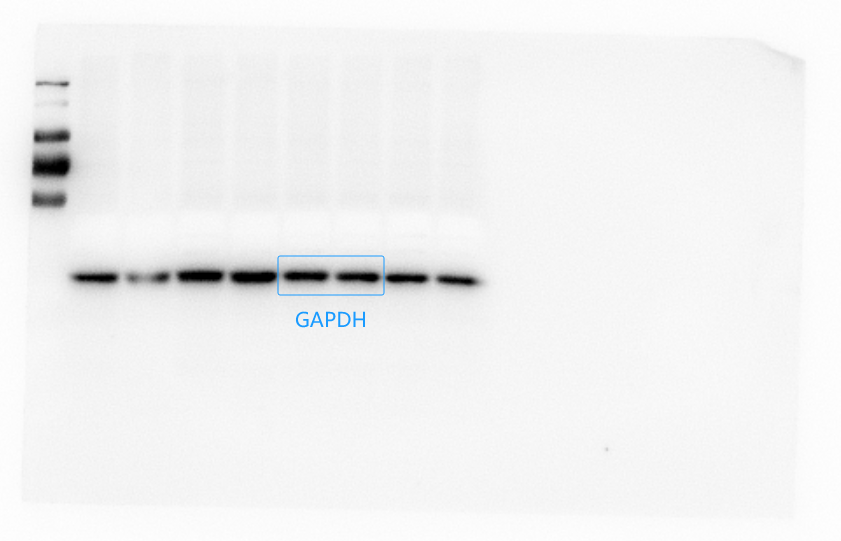


Figure 6a


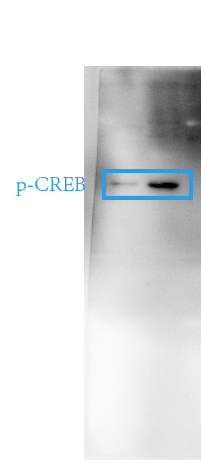

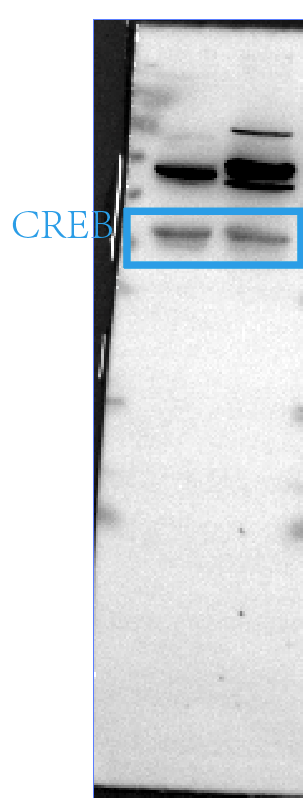

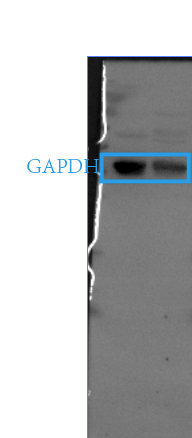


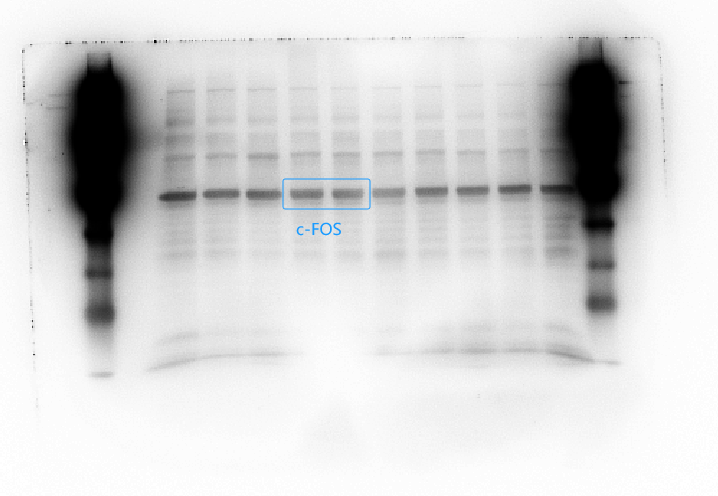


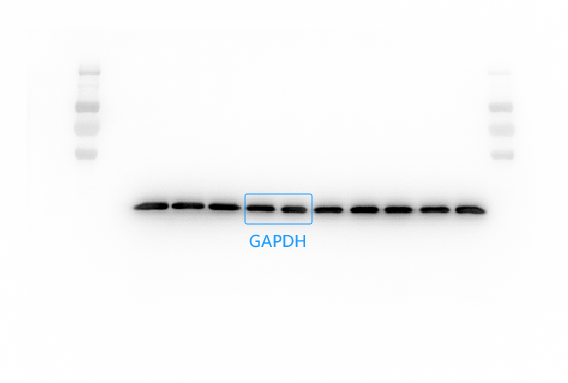


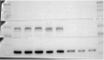


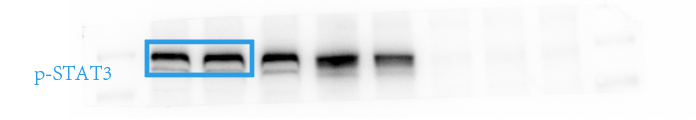

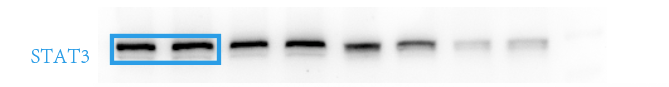


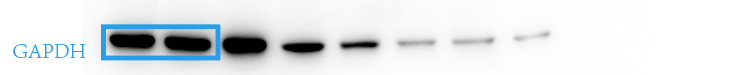


Figure 6b


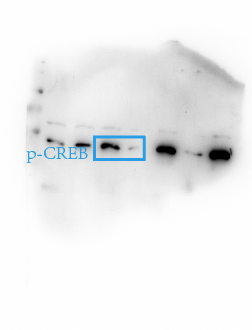


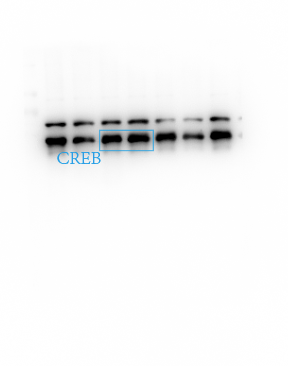


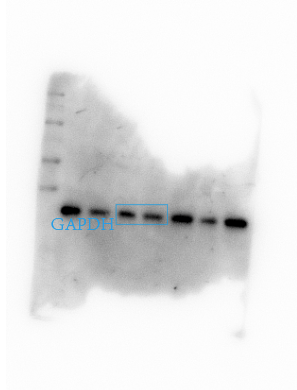


Figure 6c


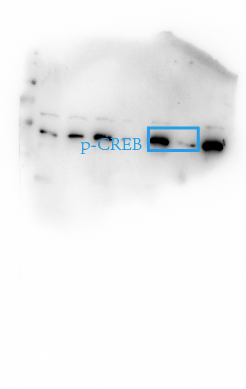

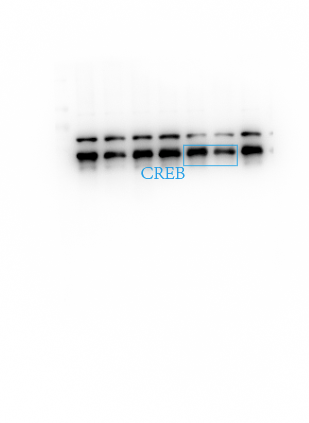


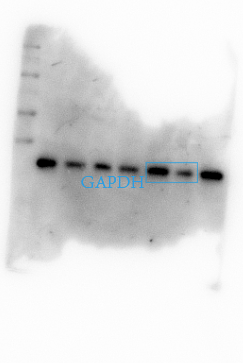


Figure 6d


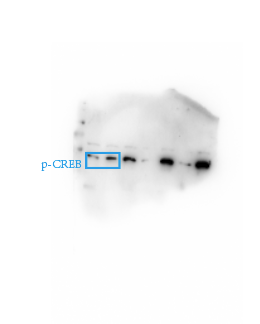


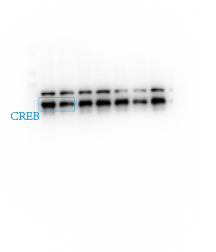


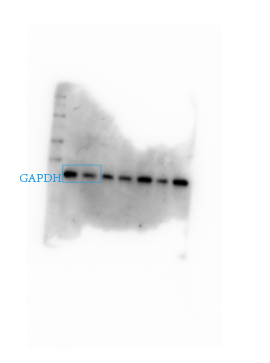


Figure 6e


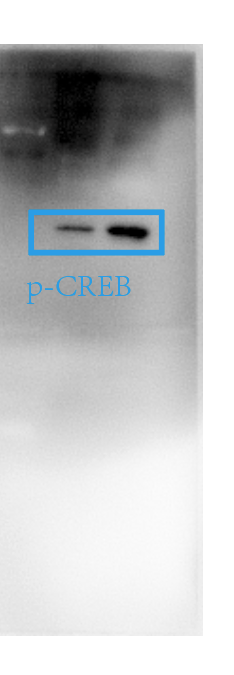

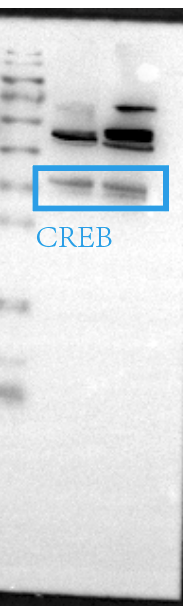

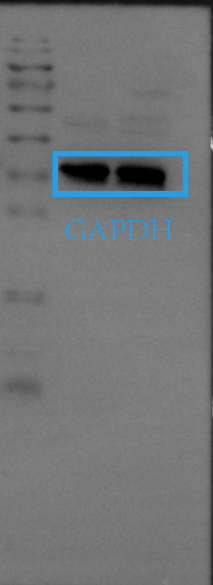


Figure 6g


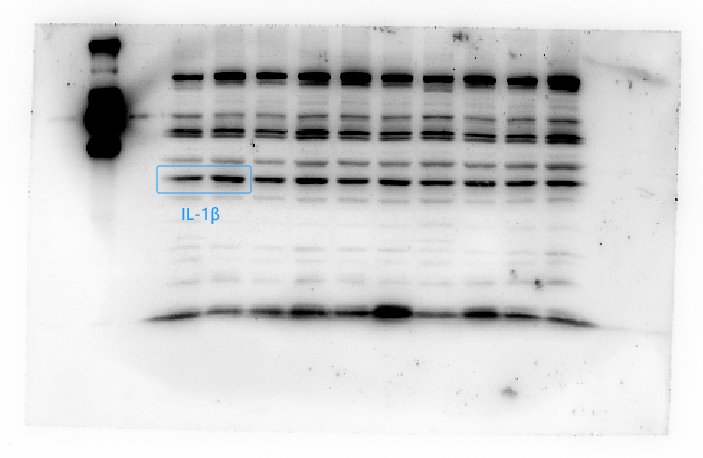


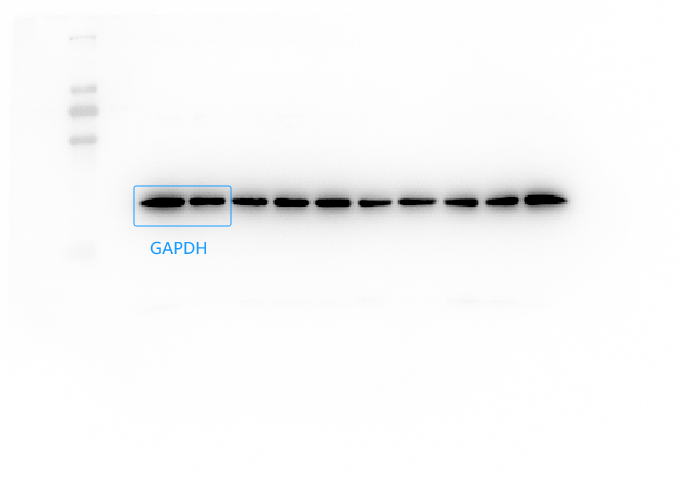


Figure 6i


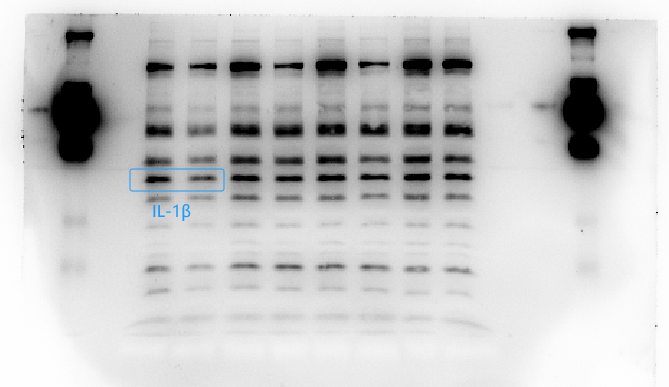


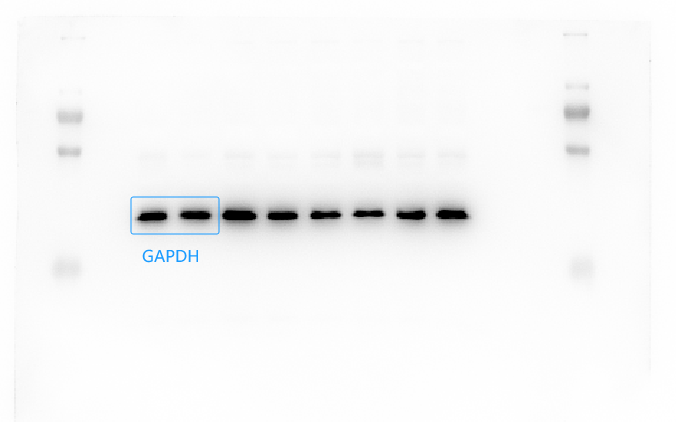


Figure 6k


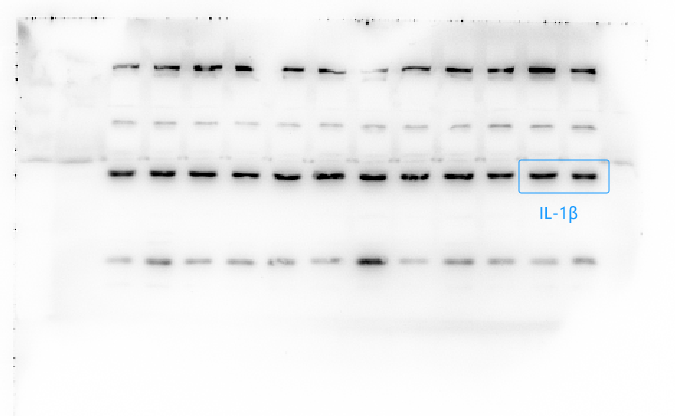


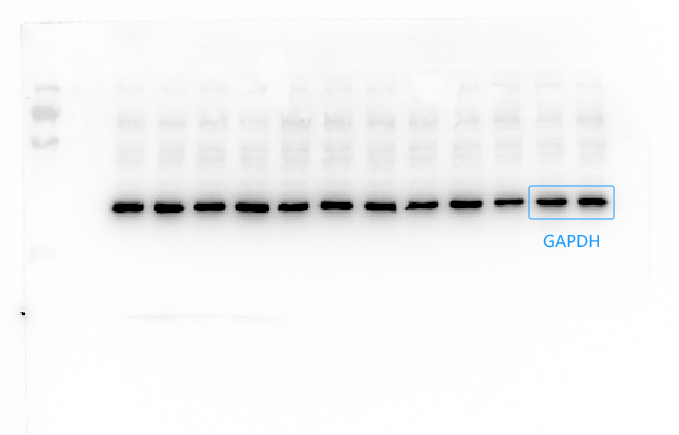


Figure 6m


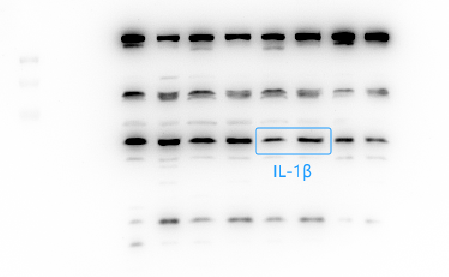


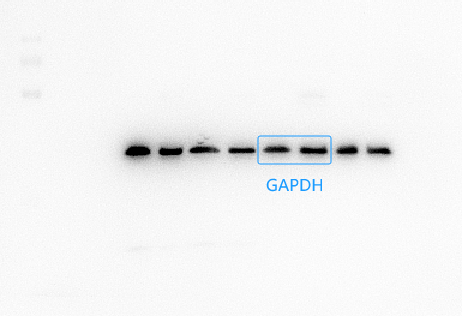


Figure 6o


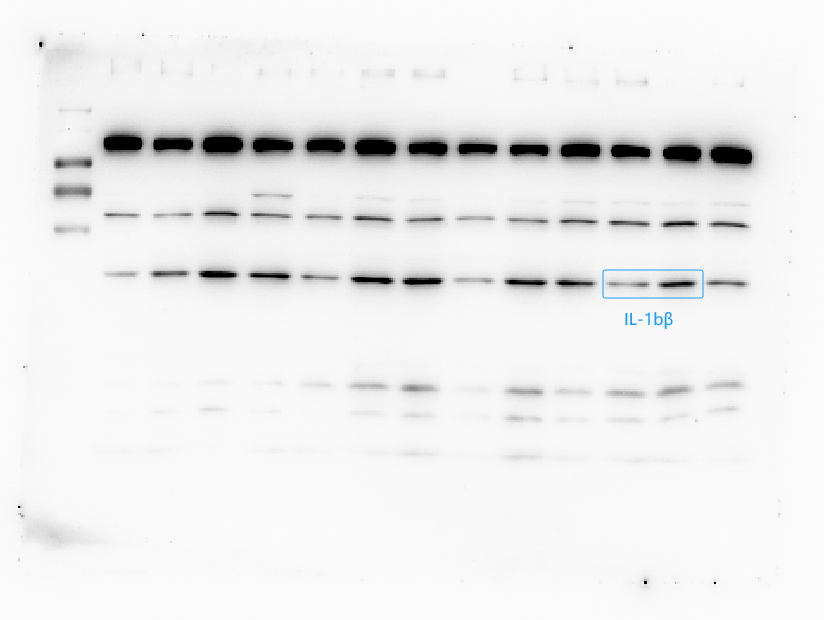


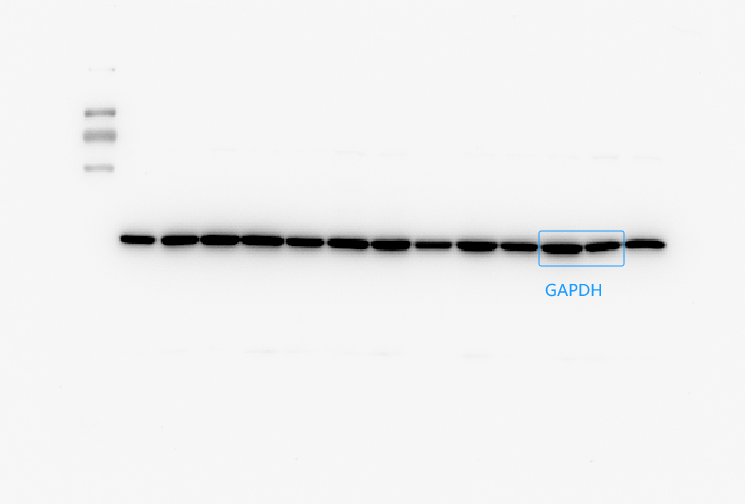


Figure 6q


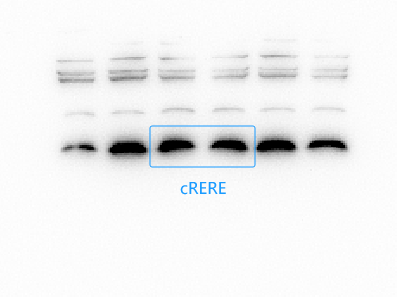


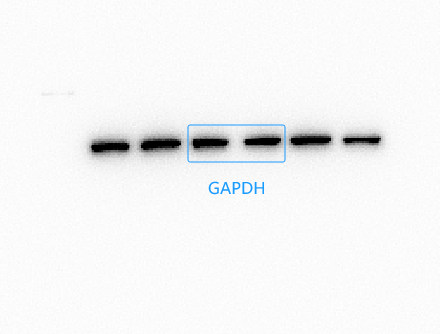


Figure S4b


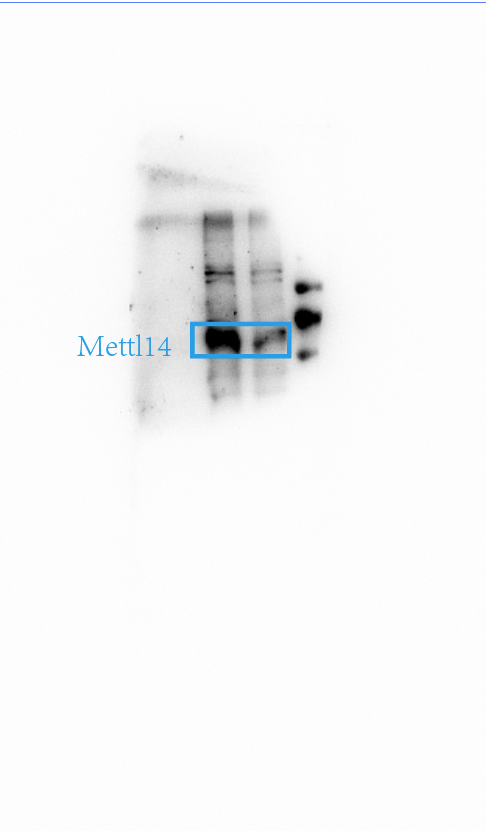

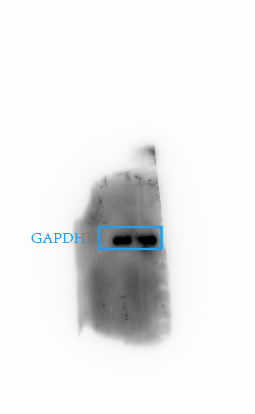

Supplement: Supplementary file 8 — Supplementary Material 8. [file 12964_2025_2455_MOESM8_ESM.docx]
