# Supplementary figures and images for: A novel protein cRERE encoded by a circular RNA directly targets ERK signaling to alleviate chemotherapy-induced neuropathic pain
Source: Cell Commun Signal. 2025 Oct 17;23:445. doi: 10.1186/s12964-025-02455-x (PMC12535093; doi:10.1186/s12964-025-02455-x)

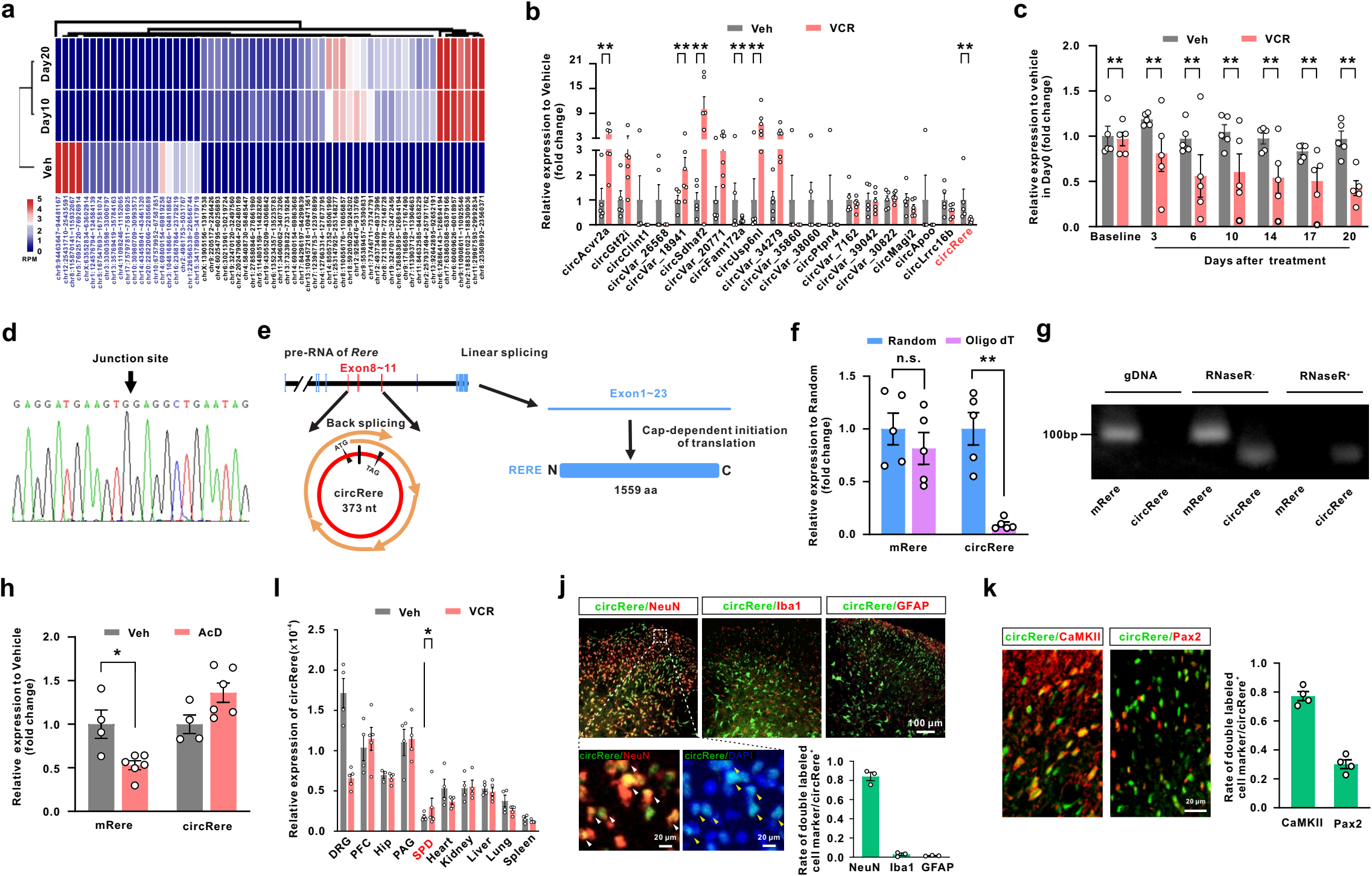

Supplement: Supplementary file 9 — The original format of Figure 1 [file 12964_2025_2455_MOESM9_ESM.pdf]

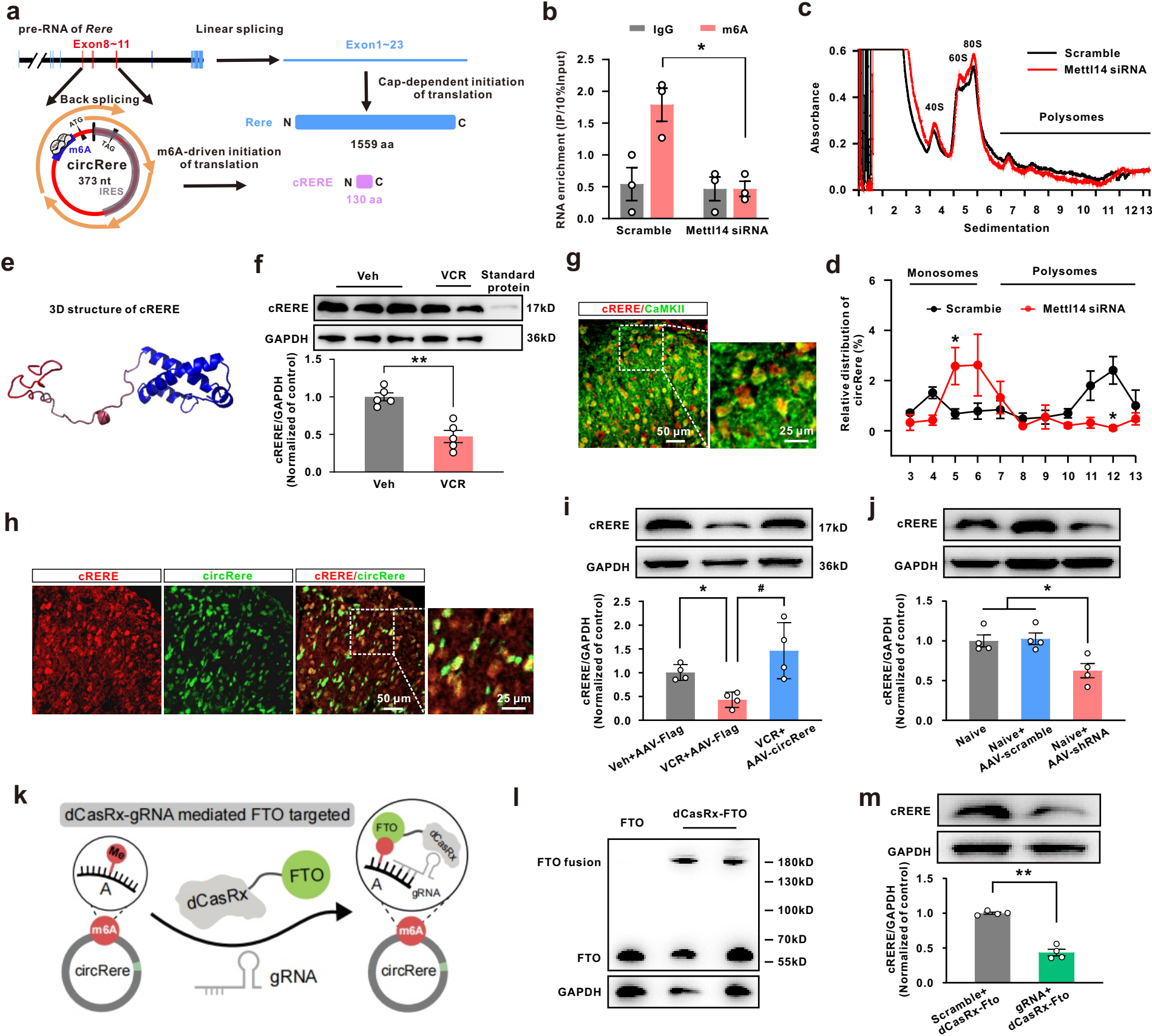

Supplement: Supplementary file 10 — The original format of Figure 2 [file 12964_2025_2455_MOESM10_ESM.pdf]
